# Nasopharyngeal fungal subtypes of infant bronchiolitis and disease severity risk

**DOI:** 10.1016/j.ebiom.2023.104742

**Published:** 2023-08-01

**Authors:** Ryohei Shibata, Zhaozhong Zhu, Michihito Kyo, Tadao Ooka, Robert J. Freishtat, Jonathan M. Mansbach, Marcos Pérez-Losada, Carlos A. Camargo, Kohei Hasegawa

**Affiliations:** a Department of Emergency Medicine, Massachusetts General Hospital, Harvard Medical School, Boston, MA, USA; b Department of Health Science, University of Yamanashi, Chuo, Yamanashi, Japan; c Center for Genetic Medicine Research, Children’s National Research Institute, Washington, DC, USA; d Division of Emergency Medicine, Children’s National Hospital, Washington, DC, USA; e Department of Pediatrics, The George Washington University School of Medicine and Health Sciences, Washington, DC, USA; f Department of Pediatrics, Boston Children’s Hospital, Harvard Medical School, Boston, MA, USA; g Computational Biology Institute, Department of Biostatistics and Bioinformatics, The George Washington University, Washington, DC, USA

**Keywords:** Bronchiolitis, Fungi, Metatranscriptome, Mycotype, Severity, Transcriptome

## Abstract

**Background:**

Bronchiolitis is a leading cause of infant hospitalization. Recent research suggests the heterogeneity within bronchiolitis and the relationship of airway viruses and bacteria with bronchiolitis severity. However, little is known about the pathobiological role of fungi. We aimed to identify bronchiolitis mycotypes by integrating fungus and virus data, and determine their association with bronchiolitis severity and biological characteristics.

**Methods:**

In a multicentre prospective cohort study of 398 infants (age <1 year, male 59%) hospitalized for bronchiolitis, we applied clustering approaches to identify mycotypes by integrating nasopharyngeal fungus (detected in RNA-sequencing data) and virus data (respiratory syncytial virus [RSV], rhinovirus [RV]) at hospitalization. We examined their association with bronchiolitis severity—defined by positive pressure ventilation (PPV) use and biological characteristics by nasopharyngeal metatranscriptome and transcriptome data.

**Results:**

In infants hospitalized for bronchiolitis, we identified four mycotypes: **A)** fungi^*M.restricta*^virus^RSV/RV^, **B)** fungi^*M.restricta*^virus^RSV^, **C)** fungi^*M.globosa*^virus^RSV/RV^, **D)** fungi^not-detected^virus^RSV/RV^ mycotypes. Compared to mycotype A infants (the largest subtype, n = 211), mycotype C infants (n = 85) had a significantly lower risk of PPV use (7% vs. 1%, adjOR, 0.21; 95% CI, 0.02–0.90; p = 0.033), while the risk of PPV use was not significantly different in mycotype B or D. In the metatranscriptome and transcriptome data, mycotype C had similar bacterial composition and microbial functions yet dysregulated pathways (e.g., Fc γ receptor-mediated phagocytosis pathway and chemokine signaling pathway; FDR <0.05).

**Interpretation:**

In this multicentre cohort, fungus-virus clustering identified distinct mycotypes of infant bronchiolitis with differential severity risks and unique biological characteristics.

**Funding:**

This study was supported by the 10.13039/100000002National Institutes of Health.

**Research in context**

**Evidence before this study**

Bronchiolitis is a leading cause of infant hospitalization in the U.S. Recent research suggests the heterogeneity within bronchiolitis and the relationship of airway viruses and bacteria with bronchiolitis severity. However, little is known about the pathobiological role of fungi and the exact mechanisms underlying the heterogeneity-severity link. We searched PubMed for studies published from inception to December 2022, with the search terms “bronchiolitis”, or “severity”, or “fungi”, or “*Malassezia*”, or “respiratory syncytial virus”, or “rhinovirus”, or “bacteria”, or “subtype”, or “mycotype”, or “transcriptome”, or “metatranscriptome” with no language restrictions. No study has reported on fungus-virus subtypes (mycotypes) of infant bronchiolitis and their disease severity and biological characteristics.

**Added value of this study**

By applying clustering approaches to fungus and virus data from a prospective multicentre cohort study of infants hospitalized for bronchiolitis, we identified four clinically meaningful mycotypes. Infants with a mycotype characterized by the highest *Malassezia globosa* abundance and respiratory syncytial virus and rhinovirus infection had the lowest bronchiolitis severity—defined by positive pressure ventilation use. Additionally, although infants with this mycotype had no distinct bacterial composition and microbial functions in the nasopharyngeal metatranscriptome data, the infants had distinct host airway response (e.g., Fc γ receptor-mediated phagocytosis pathway and chemokine signaling pathway) in the nasopharyngeal host transcriptome data.

**Implications of all the available evidence**

Our findings based on mycotypes with different bronchiolitis severity risks and distinct biological characteristics offer an evidence-base for developing potential prophylactic and therapeutic strategies (e.g., modulating host immune response by altering airway fungal profile) against infant bronchiolitis.

## Introduction

Bronchiolitis—the most common lower respiratory infection and leading cause of hospitalization among infants in the U.S.—is an important health problem.^1^^,^^2^ The severity varies from a minor nuisance to fatal infection.^2^, ^3^, ^4^ While bronchiolitis has traditionally been considered a single disease with a similar mechanism,^5^ growing evidence supports heterogeneity within bronchiolitis.^6^, ^7^, ^8^, ^9^, ^10^ Indeed, recent studies have reported subtypes of infant bronchiolitis with different severity risks according to respiratory virus^6^^,^^8^^,^^9^ and upper airway microbiome (more specifically, the bacteriome)^10^ data. Yet, the exact mechanisms underlying the heterogeneity-severity link are still mostly unknown.

In addition to viruses and bacteria, emerging evidence has indicated that fungi are common members in the airway^11^, ^12^, ^13^, ^14^ and play protective or pathological roles not only in host immunity but also in airway diseases. For example, recent studies have reported that airway fungi were captured by antigen presentation cells and induced Th17-neutrophil immunity in both mouse^15^, ^16^, ^17^, ^18^ and human lung tissue.^19^ The literature has also shown the association of airway fungal profile with airway diseases among adults (e.g., chronic rhinosinusitis,^20^^,^^21^ cystic fibrosis,^21^ and asthma^14^^,^^22^, ^23^, ^24^, ^25^, ^26^). Within the sparse literature in children, single-centre data of infants with respiratory syncytial virus (RSV) infection (n = 43) have reported an association between a higher abundance of *Malassezia globosa* (commensal yeast) and a lower risk of hospitalization.^12^ Thus, despite the potential for clinical and research significance, little is known about the role of fungi in respiratory infection—let alone in infants with bronchiolitis.

To address this knowledge gap, we analysed data from a multicentre cohort study of infants hospitalized for bronchiolitis to: 1) identify subtypes using fungus and virus data (mycotypes), 2) investigate their associations with bronchiolitis severity, and 3) determine their biological significance by integrating nasopharyngeal metatranscriptome and host transcriptome data. A better understanding of mycotypes may inform early-life prophylactic and therapeutic strategies for bronchiolitis.

## Methods

### Study design, setting, and participants

We analysed data from the 35th Multicenter Airway Research Collaboration (MARC-35) study—a multicentre prospective cohort study. Details of the study design, setting, participants, data collection, testing, and statistical analysis may be found in the Supplementary Methods. Briefly, investigators enrolled infants (age <1 year) hospitalized with attending physician-diagnosis of bronchiolitis at 17 sites across 14 U.S. states (Supplementary Table S1) in 2011–2014. Bronchiolitis was defined by the American Academy of Pediatrics (AAP) guidelines—acute respiratory illness with some combination of rhinitis, cough, tachypnoea, wheezing, crackles, and retractions, regardless of previous breathing problem episodes.^27^ We excluded infants with a known heart-lung disease, immunodeficiency, immunosuppression, or gestational age <32 weeks. All patients were treated at the discretion of the treating physician.

### Ethics

This study that involves human participants was conducted in accordance with the Declaration of Helsinki and approved by the Human Research Committee at Massachusetts General Hospital (protocol 2017P001861). Participant’s caregiver gave informed consent to participate in the study before taking part.

### Data collection

Clinical data (patients’ demographic characteristics, medical history, environmental, and family, and details of the acute illness) were collected via structured interview and chart reviews.^10^ All data were reviewed at the EMNet Coordinating Center at Massachusetts General Hospital (Boston, MA, U.S.A.), and site investigators were queried about missing data and discrepancies identified by manual data checks. Nasopharyngeal specimens were collected within 24 h of hospitalization using standard protocols.^7^^,^^10^ Nasopharyngeal specimens were tested for respiratory viruses (e.g., RSV and rhinovirus [RV]) by real-time polymerase chain reaction assays,^7^^,^^10^^,^^28^ metatranscriptome and host transcriptome profiles by RNA-sequencing (RNA-seq).

### Nasopharyngeal total RNA extraction, RNA-seq, and metatranscriptome and host transcriptome profiling

The details of RNA extraction, RNA-seq, and quality control in the metatranscriptome and host transcriptome data are described in the Supplementary Methods and our previous studies.^29^, ^30^, ^31^ Briefly, from 398 randomly selected nasopharyngeal specimens, after total RNA extraction, DNase treatment, and rRNA reduction, we performed RNA-seq with a NovaSeq6000 (Illumina, San Diego, CA, U.S.A.) using an S4 100bp PE Flowcell (Illumina). All RNA-seq samples had high sequence coverage (mean, 48,014,679 pair-end reads/sample).

For metatranscriptome profiling, after the quality control, we first inferred fungal taxonomy by EukDetect^32^ using the EukDetect marker database built on January 22, 2022 (available from: https://figshare.com/articles/dataset/Eukdetect_database/12670856/8) and default settings. We also calculated reads per kilobase of sequence (RPKS; i.e., absolute abundance) and the relative abundance (EukFrac). Second, we inferred bacterial taxonomy by MetaPhlAn 3.0^33^ using the ChocoPhlAn database v30.^33^ Third, we conducted microbial functional profiling. The reads were aligned to UniRef90 by DIAMOND,^34^ annotated into MetaCyc metabolic pathways^35^ by HUMAnN 3.0,^33^ and mapped against this database to quantify pathway presence and abundance. Lastly, we normalized bacterial composition and microbial function raw values to relative abundance by total-sum scaling.

For host transcriptome profiling, after the quality control, we first estimated transcript abundances in Salmon^36^ using the human genome (hg38) and the mapping-based mode. Next, by calculating log10-transformed total read count per specimen, we filtered the 14 mRNA data with the low read count defined as less than the mean minus 2.5 standard deviations. Lastly, we normalized the read count by the R *DESeq2* package^37^ using default settings.

### Outcome measure

The clinical outcome of interest was acute severity of bronchiolitis, which varies in a hospitalized cohort from a short (<24-h) uneventful stay to endotracheal intubation. Specifically, for this analysis, the primary outcome was the use of positive pressure ventilation (PPV), defined as the use of continuous positive airway pressure ventilation and/or mechanical ventilation during the index hospitalization.^38^ The secondary outcome was a hospital length of stay (LOS) of ≥2 days, defined using the median LOS of 2 days.

### Statistical analysis

**Fig. 1:**
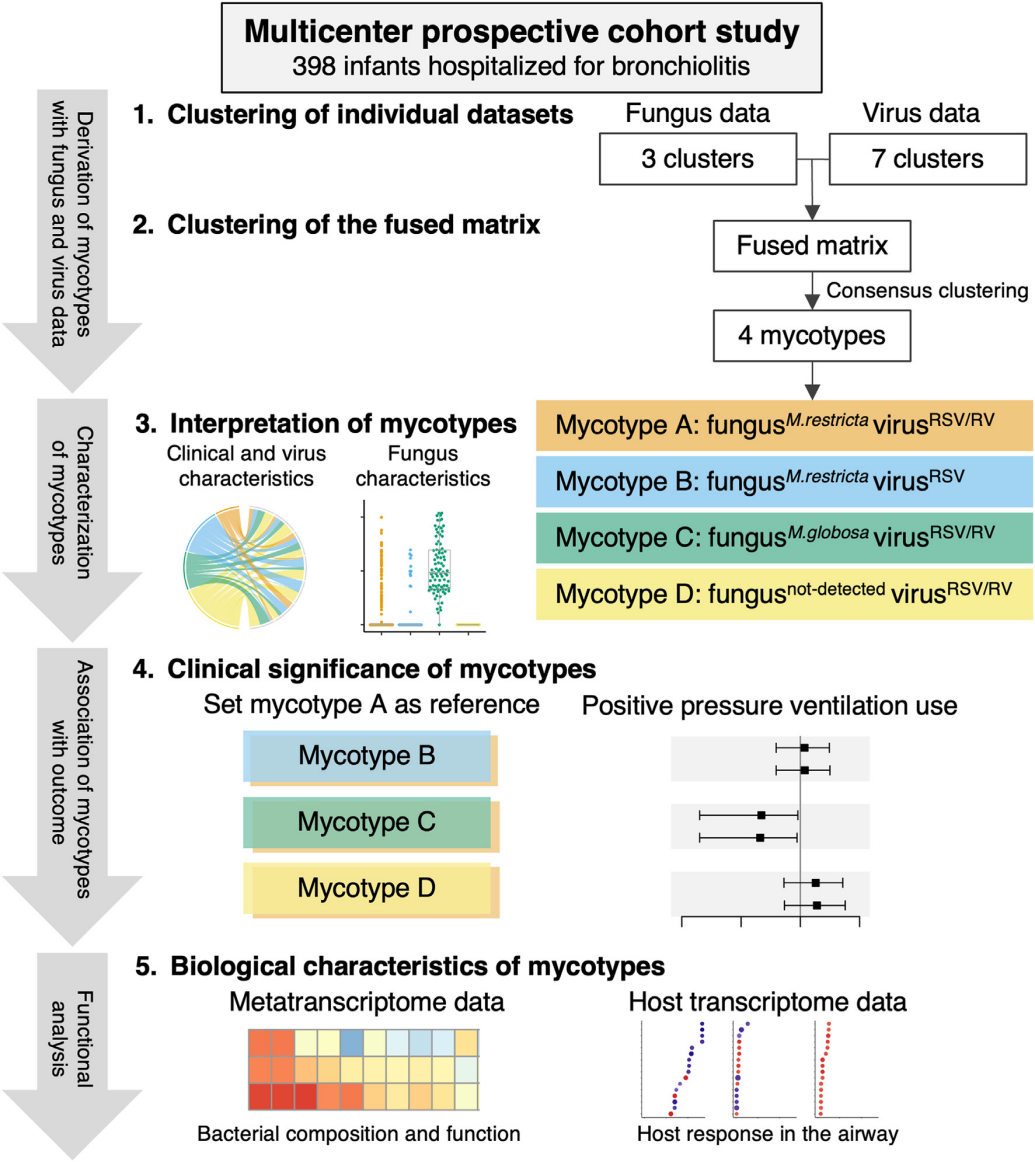
**Analytic workflow of mycotyping analysis. 1. Clustering of individual datasets:** By using multicentre prospective cohort (MARC-35) data of 398 infants (age <1 year) hospitalized for bronchiolitis, we first identified mutually exclusive clusters for each of the fungus and virus datasets. We generated three fungus clusters, including two clusters by using a Dirichlet multinomial mixtures model and one cluster consisting of infants without detectable fungi. Next, we identified seven virus clusters by computing a Gower distance, followed by applying a consensus clustering algorithm to the distance matrix. **2. Clustering of the fused matrix:** By integrating these derived clusters from the fungus and virus datasets, we generated a fused matrix and computed a Gower distance. We identified four mutually exclusive mycotypes by applying a consensus clustering algorithm to the distance matrix. To select an optimal number of profiles, we used a combination of consensus matrix, consensus cumulative distribution function, and cluster consensus value, in addition to the mycotype size and biological plausibility. **3. Interpretation of mycotypes:** To interpret the clinical, virus, and fungus characteristics of the four mycotypes, we developed chord diagrams on major clinical and virus factors and bee swarm plots on fungus factors. **4. Clinical significance of mycotypes:** To examine the clinical significance of the four mycotypes, we determined the relationship of the mycotypes with the risk of higher disease severity (defined by positive pressure ventilation use) by constructing logistic regression models. **5. Biological characteristics of mycotypes:** To examine the difference in biological characteristics between the mycotypes, we first compared bacterial composition and microbial functions by using nasopharyngeal metatranscriptome data. Next, we compared host response in the airway by a functional pathway analysis using nasopharyngeal host transcriptome data. Abbreviations: RSV, respiratory syncytial virus; RV, rhinovirus.

The analytic workflow is summarized in Fig. 1. The details of the statistical analysis can be found in the Supplementary Methods. Briefly, we first identified mutually exclusive clusters for each of the fungus and virus datasets collected at the index hospitalization. For the fungus data where at least one fungal species was detected, we used the relative abundance data of nasopharyngeal fungal species and derived the clusters by a Dirichlet multinomial mixtures (DMM) model.^39^ To choose an optimal number of these clusters, we used a Laplace approximation to the negative log model evidence. Additionally, we grouped one cluster by the fungus data without detectable fungi, added this cluster to the clusters based on a DMM model, and generated the fungus clusters. For the virus data (including the genomic load [i.e., cycle threshold value] of RSV and RV), we computed a Gower distance and derived the virus clusters by using a consensus clustering algorithm^40^ with partitioning around medoids (PAM) method. To choose an optimal number of the virus clusters, we used a combination of separations of the consensus matrix, consensus cumulative distribution function (CDF), and cluster consensus value, rather than using the indices separately, in addition to the cluster size and clinical plausibility.^6^, ^7^, ^8^ Second, we combined the fungus and virus clusters to derive a fused matrix, computed a Gower distance, and identified mutually exclusive four mycotypes by using a consensus clustering algorithm with PAM method. Third, to interpret the characteristics of the mycotypes, we developed chord diagrams on the relationship of the mycotypes with major clinical and virus variables. Additionally, to interpret the fungus characteristics, we developed bee swarm plots by the Shannon index as well as the relative and absolute abundance. Fourth, to determine the association of the mycotypes (the exposure) with the risk of PPV use and a LOS ≥2 days (the outcomes), we first constructed a directed acyclic graph to represent our causal structure hypothesis, along with potential confounders (i.e., age and use of systemic corticosteroids for breathing problems that caused the index hospitalization). The potential confounders were selected based on clinical plausibility, *a priori* knowledge,^11^^,^^12^^,^^41^^,^^42^ and the number of patients with the outcome. Next, we constructed unadjusted and multivariable logistic regression models. Regarding the association with the risk of PPV use, the models were corrected by Firth’s penalized method to reduce a bias of maximum likelihood estimate for a low proportion of the outcome.^43^ In the multivariable models, we adjusted for the potential confounders. Fifth, to examine the differences in biological characteristics in nasopharyngeal bacterial composition and microbial functions between the derived mycotypes, by the metatranscriptome data, we created heatmaps of the Shannon index and relative abundance and tested the differences using the Wilcoxon rank-sum test. Additionally, to examine the relationships of the bacterial composition and microbial functions with the outcomes, by using the relative abundance and the binary outcome data, we summarized Spearman’s rho in the heatmaps. Lastly, to examine the between-mycotype difference in biological characteristics in host airway response, by the host transcriptome data, we conducted differential expression gene analysis. We also conducted Gene Set Enrichment Analysis^44^^,^^45^ based on Environmental Information Processing and Organismal Systems in Kyoto Encyclopedia of Genes and Genomes PATHWAY^46^ and descendants of GO:0002376 immune system process in Biological Processes of Gene Ontology.

In the sensitivity analysis, we first computed E-values to determine the robustness of causal inference to potential unmeasured confounding. Next, we confirmed the mycotype-outcome associations by constructing different models. For example, considering that Firth’s penalized method could lead to artifacts (e.g., the estimates fall outside the range of the prior median and the maximum likelihood estimate), we constructed logistic regression models with the log*F*-type penalized method.^47^^,^^48^ We also estimated risk ratios by constructing modified Poisson regression models (i.e., Poisson regression models followed by sandwich error estimation).^49^ Additionally, we examined the mycotype-outcome associations after excluding infants with a previous history of breathing problem. Lastly, we examined the robustness of the mycotype-outcome associations by repeating the analysis using a different number of mycotypes.

Analysis used R version 4.1.3 (R Foundation, Vienna, Austria). All p-values were two-tailed, with p < 0.05 considered statistically significant. We computed the Benjamin-Hochberg FDR that allows for the interpretation of statistical significance in the context of multiple hypothesis testing,^50^ with FDR <0.05 considered statistically significant.

### Role of the funders

The funding organization was not involved in the collection, management, or analysis of the data; preparation or approval of the manuscript; or decision to submit the manuscript for publication.

## Results

Of the 1016 infants enrolled into the MARC-35 cohort, the current study focused on 398 infants with the nasopharyngeal RNA-seq data at enrolment. The analytic and non-analytic cohorts did not differ in patient characteristics (p ≥ 0.05; Supplementary Table S2), except for the genomic load of RSV. Among the analytic cohort, the median age was 3 (interquartile range 2–7) months, 59% were male, and 40% were non-Hispanic white. Overall, 80% had RSV, 22% had RV, and 6% underwent PPV (Table 1 and Supplementary Table S3). For fungal taxonomic profiling in the nasopharyngeal airway, a total of 30 fungal species were identified, and no fungi were detected in 38 infants (10%). The two most abundant fungal species were *Malassezia restricta* and *M. globosa* (Supplementary Table S4). For bacterial taxonomic profiling, a total of 268 bacterial species were identified. For microbial functional profiling, an average of 65% of all RNA reads were assigned to UniRef90 gene families, and the unique gene families were assigned to a total of 368 unique microbial functional pathways.

**Table 1:** Baseline patient characteristics and clinical course of infants hospitalized for bronchiolitis, according to four mycotypes.

Variables	Overall (n = 398; 100%)	Mycotype A (fungus^*M.restricta*^ virus^RSV/RV^) (n = 212; 53%)	Mycotype B (fungus^*M.restricta*^ virus^RSV^) (n = 64; 16%)	Mycotype C (fungus^*M.globosa*^ virus^RSV/RV^) (n = 87; 22%)	Mycotype D (fungus^Not-detected^ virus^RSV/RV^) (n = 35; 9%)	p value^a^
Demographics						
Age, month						0.95
<2	120 (30)	64 (30)	19 (30)	25 (29)	12 (34)	
2–5	139 (35)	76 (36)	22 (34)	32 (37)	9 (26)	
5–11.9	139 (35)	72 (34)	23 (36)	30 (34)	14 (40)	
Male sex	233 (59)	123 (58)	36 (56)	47 (54)	27 (77)	0.094
Race/ethnicity						0.85
Non-Hispanic white	161 (40)	83 (39)	25 (39)	39 (45)	14 (40)	
Non-Hispanic black	91 (23)	55 (26)	13 (20)	18 (21)	5 (14)	
Hispanic	129 (32)	64 (30)	23 (36)	27 (31)	15 (43)	
Other	17 (4)	10 (5)	3 (5)	3 (3)	1 (3)	
C-section delivery	138 (35)	71 (33)	25 (39)	31 (36)	11 (31)	0.84
Prematurity (32–36.9 weeks)	72 (18)	43 (20)	12 (19)	14 (16)	3 (9)	0.39
History of eczema	61 (15)	36 (17)	8 (12)	13 (15)	4 (11)	0.80
Previous breathing problems (count)						0.86
0	314 (79)	167 (79)	51 (80)	67 (77)	29 (83)	
1	62 (16)	33 (16)	10 (16)	16 (18)	3 (9)	
≥2	22 (6)	12 (6)	3 (5)	4 (5)	3 (9)	
Ever attended daycare	95 (24)	54 (25)	15 (23)	17 (20)	9 (26)	0.73
Cigarette smoke exposure at home	60 (15)	28 (13)	14 (22)	13 (15)	5 (14)	0.42
Mostly breastfed during 0–2.9 months	166 (42)	82 (39)	29 (45)	42 (48)	13 (37)	0.80
Lifetime antibiotics use	126 (32)	68 (32)	18 (28)	31 (36)	9 (26)	0.70
Lifetime corticosteroid use^c^	62 (16)	29 (14)	12 (19)	19 (22)	2 (6)	0.093
Recent corticosteroid use^d^	42 (11)	21 (10)	7 (11)	14 (16)	0 (0)	0.048
Maternal smoking during pregnancy	62 (16)	35 (17)	10 (16)	15 (17)	2 (6)	0.42
Clinical presentation at index hospitalization						
Weight (kg), median (IQR)	6.2 (4.7–8.0)	6.1 (4.8–7.9)	6.2 (4.9–8.1)	6.3 (4.7–7.9)	6.3 (4.4–8.6)	0.94^b^
Respiratory rate (per minute), median (IQR)	50 (40–60)	48 (40–60)	48 (40–60)	52 (40–60)	52 (43–60)	0.94^b^
Oxygen saturation						0.26
<90%	37 (10)	21 (10)	6 (9)	9 (11)	1 (3)	
90–93.9%	56 (14)	28 (13)	9 (14)	8 (9)	11 (31)	
≥94%	294 (74)	156 (74)	47 (73)	68 (78)	23 (66)	
Fungi clusters						0.00050
Cluster 1 (*M. restricta* dominant)	273 (69)	212 (100)	61 (95)	0 (0)	0 (0)	
Cluster 2 (*M. globosa* dominant)	87 (22)	0 (0)	0 (0)	87 (100)	0 (0)	
Cluster 3 (infants without detectable fungi)	38 (10)	0 (0)	3 (5)	0 (0)	35 (100)	
Viral testing						
RSV	317 (80)	158 (75)	64 (100)	70 (80)	25 (71)	0.00050
Solo-RSV	225 (57)	107 (50)	51 (80)	53 (61)	14 (40)	0.00050
RSV cycle threshold values, median (IQR)	22 (20–25)	21 (19–22)	26 (25–30)	22 (20–24)	21 (19–21)	<0.0001^b^
RV	86 (22)	58 (27)	0 (0)	19 (22)	9 (26)	0.00050
Non-RSV and non-RV	42 (11)	25 (12)	0 (0)	8 (9)	9 (26)	0.00050
Other pathogens^e^	99 (25)	62 (29)	12 (18)	14 (16)	11 (31)	0.040
Clinical course						
Received antibiotics during pre-hospitalization visit	68 (17)	39 (18)	15 (23)	9 (10)	5 (14)	0.16
Received corticosteroids during pre-hospitalization visit	37 (9)	24 (11)	6 (9)	5 (6)	2 (6)	0.47
Positive pressure ventilation use^f^	25 (6)	15 (7)	5 (8)	1 (1)	4 (11)	0.054
Intensive treatment use^g^	71 (18)	30 (14)	13 (20)	18 (21)	10 (29)	0.13
Hospital length of stay (days), median (IQR)	2 (1–3)	2 (1–3)	2 (2–3)	2 (1–3)	2 (1–4)	0.24^b^
Hospital length of stay ≥2 days	265 (67)	139 (66)	50 (78)	51 (59)	25 (71)	0.073

Data are the number (percentage) of children unless otherwise indicated. Percentages may not equal 100 because of rounding and missingness. All data are collected, unless otherwise indicated.

Abbreviations: IQR, interquartile range; RSV, respiratory syncytial virus; RV, rhinovirus.

a Tested by the Fisher exact test, unless otherwise indicated.

b Tested by the Kruskal–Wallis test.

c Defined as the use of systemic corticosteroids before the index hospitalization.

d Defined as the use of systemic corticosteroids for breathing problems that caused the index hospitalization.

e Adenovirus, bocavirus, *Bordetella pertussis*, enterovirus, human coronavirus NL63, OC43, 229E, or HKU1, human metapneumovirus, influenza A or B virus, *Mycoplasma pneumoniae*, and parainfluenza virus 1–3.

f Defined as the use of invasive and/or non-invasive mechanical ventilation (e.g., continuous positive airway pressure ventilation) during the index hospitalization.

g Defined as the use of positive pressure ventilation and/or admission to intensive care unit.

### Integrated clustering of the fungus and virus datasets identified distinct mycotypes among infants with bronchiolitis

First, by applying a clustering approach to the fungus data (n = 360), a two-class model provided an optimal fit (Supplementary Figure S1). With the cluster consisting of infants without detectable fungi (n = 38), three fungus clusters were generated. Second, by applying a consensus clustering approach to the virus data, a seven-class model provided an optimal fit (Supplementary Figure S2). Next, by integrating these cluster data, the combination of the consensus matrix, CDF, cluster consensus value in addition to mycotype size, and biological plausibility indicated that a four-class model provided an optimal fit (Supplementary Figure S3) with the four mycotypes—called A, B, C, and D (Fig. 1). As shown in Table 1, the four distinct mycotypes were A) fungus^*M.restricta*^virus^RSV/RV^ (25%), B) fungus^*M.restricta*^virus^RSV^ (16%), C) fungus^*M.globosa*^virus^RSV/RV^ (22%), and D) fungus^not-detected^virus^RSV/RV^ (9%).

**Fig. 2:**
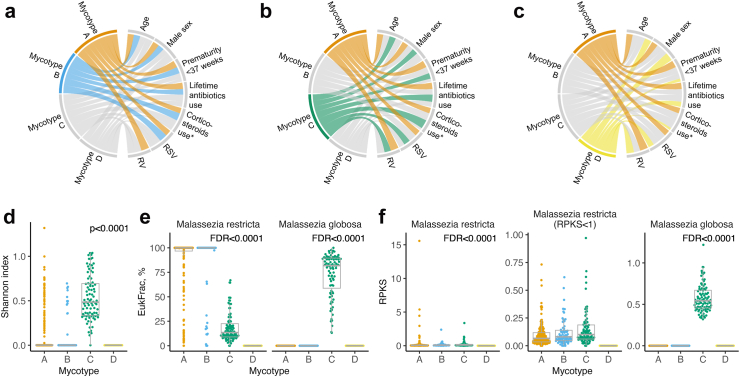
**Clinical, virus, and fungus characteristics of infants hospitalized for bronchiolitis, according to mycotypes.** To interpret clinical and virus characteristics of the four mycotypes, we constructed chord diagrams that represent the comparison between mycotypes (a) A and B, (b) A and C, and (c) A and D. Ribbons connect each of the mycotypes (mycotypes A–D) with major clinical and virus characteristics. The width of the ribbon represents the proportion of infants within the mycotypes who have the corresponding clinical or virus characteristic, which was scaled to a total of 100%. Additionally, to interpret fungus characteristics, we constructed bee swarm and box-and-whisker plots to show the distribution of (d) the Shannon index as well as (e) EukFrac (i.e., relative abundance) and (f) reads per kilobase of sequence (RPKS; i.e., absolute abundance) of the two most abundant nasopharyngeal fungal species, according to the four mycotypes. In the box-and-whisker plots, boxes and whiskers show the median and interquartile range (IQR) and 1.5 times the IQR, respectively. In the overlying bee swarm plots, each point denotes each infant, and the width represents the probability that infants in a mycotype take on a specific value. The between-mycotype differences in the Shannon index, EukFrac, and RPKS were tested by the Kruskal–Wallis test. Abbreviations: FDR, false discovery rate; RSV, respiratory syncytial virus; RV, rhinovirus. ∗Defined as the use of systemic corticosteroids for breathing problems that caused the index hospitalization.

Between the four mycotypes, the differences in clinical, virus, and fungus characteristics are summarized in Table 1 and Fig. 2. No clinical characteristics at baseline were different (p ≥ 0.05), except for the proportion of recent systemic corticosteroid use (mycotype A, B, C, and D; 10%, 11%, 16%, and 0%; p = 0.048; Table 1). Infants with a mycotype A were characterized by RSV and RV infection and a *M. restricta*-dominant fungal profile. As the mycotype A was the largest subtype (n = 212), this subtype served as the reference for the following analyses. Infants with a mycotype B were characterized by solo-RSV infection with the lower genomic load (i.e., cycle threshold value) and a *M. restricta*-dominant fungal profile. Infants with a mycotype C were characterized by the highest proportion of recent corticosteroid use, RSV and RV infection, the highest fungal Shannon index, and a *M. globosa*-dominant fungal profile. Lastly, infants with a mycotype D were characterized by no recent corticosteroid use, RSV and RV infection, and no detectable fungi.

### Mycotypes had differential disease severity risks

**Fig. 3:**
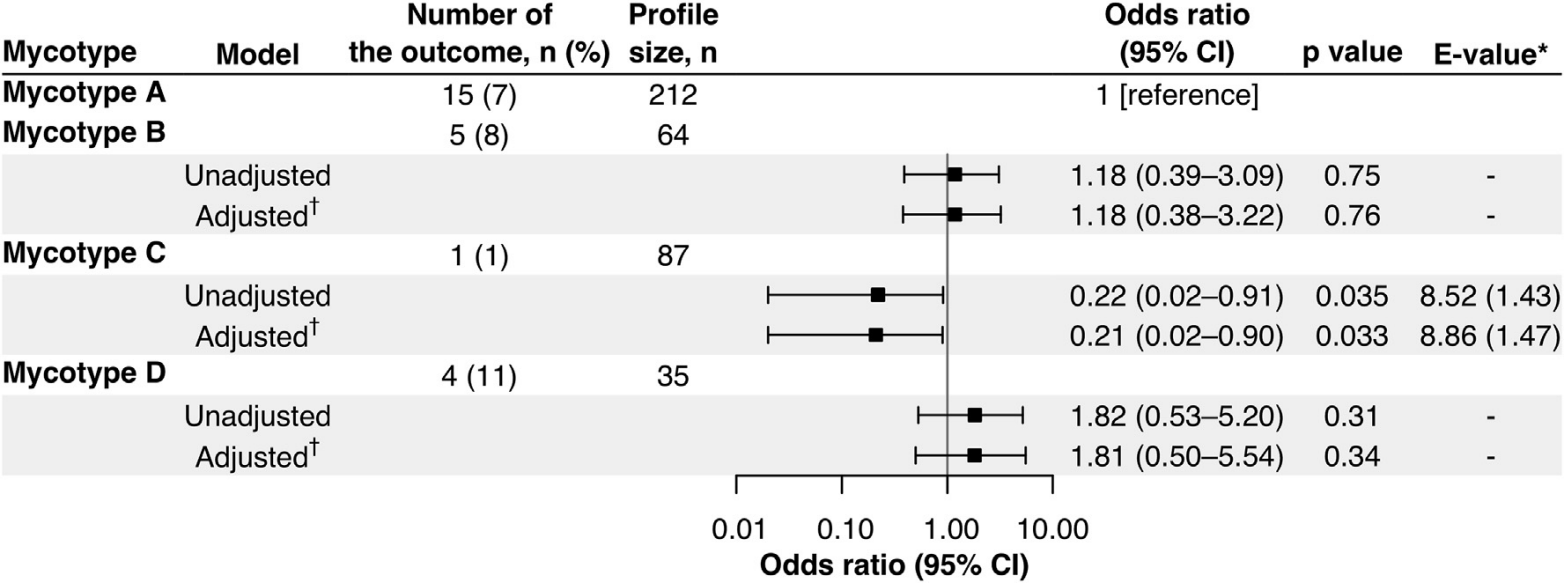
**Association of mycotypes of infant bronchiolitis with risk of positive pressure ventilation use.** To examine the association of bronchiolitis mycotypes (mycotype A as the reference) with the risk of positive pressure ventilation use, logistic regression models were constructed. The models were corrected by Firth’s penalized method to reduce a bias of maximum likelihood estimate for a low proportion of the outcome. ∗The E-value (with its value for the upper 95% confidence limit for odds ratio) represents how strongly a set of unmeasured confounders would be associated with the exposure and outcome to fully eliminate the observed association. ^†^Multivariable logistic regression model adjusted for potential confounders (i.e., age and use of systemic corticosteroids for breathing problems that caused the index hospitalization). Abbreviation: CI, confidence interval.

The identified mycotypes had differential disease severity risks (i.e., PPV use and LOS of ≥2 days, Supplementary Figure S4). Compared with mycotype A, mycotype C (fungus^*M.globosa*^virus^RSV/RV^) had a lower risk of PPV use (7% vs. 1%; adjusted odds ratio [adjOR] 0.21; 95% confidence interval [CI], 0.02–0.90; p = 0.033; E-value = 8.86; Fig. 3). In contrast, the risk of PPV use was not different in mycotype B or D (Fig. 3). Consistent with the risks of PPV use, mycotype C had the lowest risk of LOS of ≥2 days (66% vs. 59%; adjOR, 0.74; 95% CI, 0.44–1.25; p = 0.26; Supplementary Figure S5).

### Mycotypes had similar bacterial composition and microbial functions

**Fig. 4:**
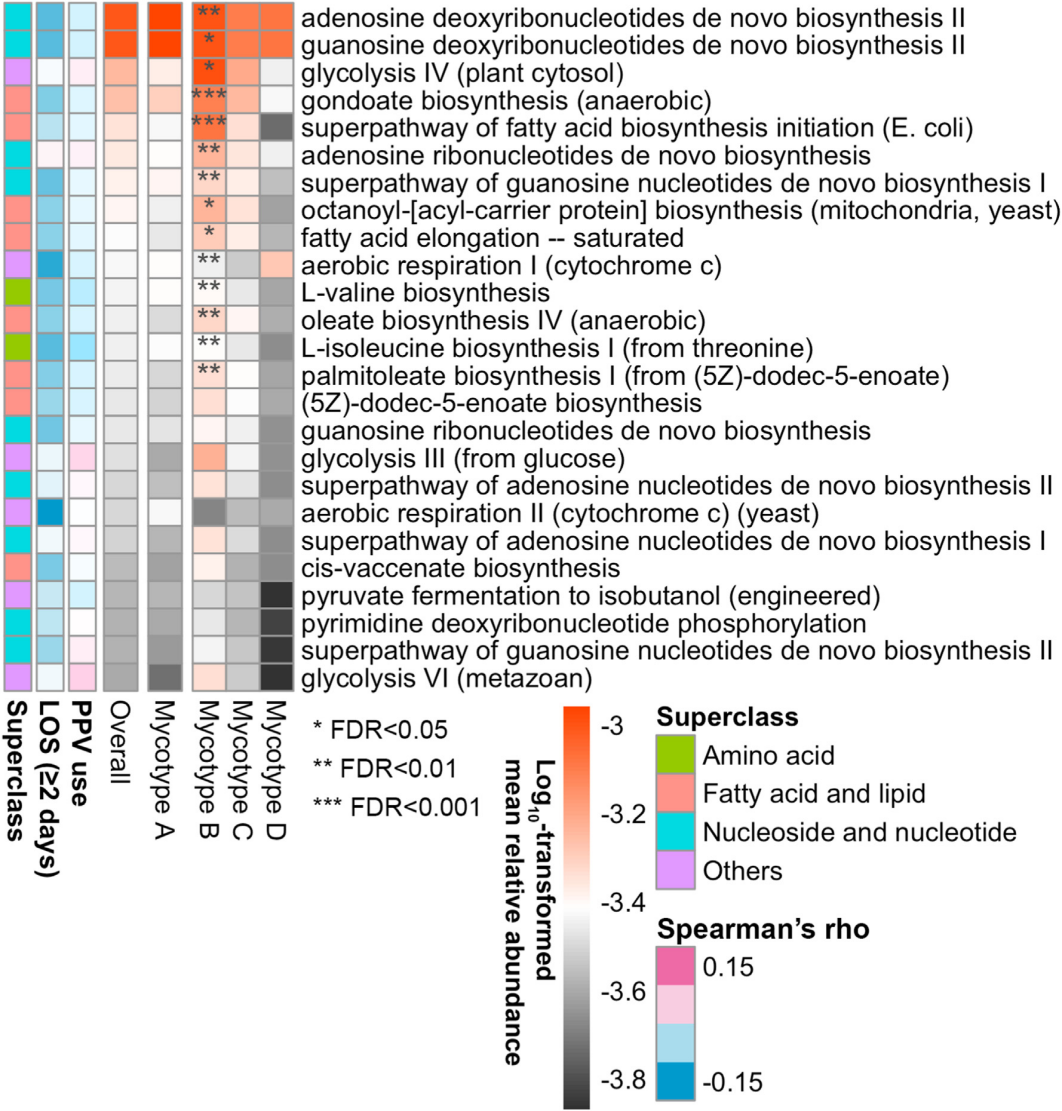
**Between-mycotype difference in relative abundance of the 25 most abundant nasopharyngeal microbial functions among infants hospitalized for bronchiolitis.** To examine the difference in the nasopharyngeal bacterial functions between mycotypes (A [the reference] vs. B, C, and D), the heatmap summarizes the relative abundance of the 25 most abundant microbial functions using the metatranscriptome data, according to the four mycotypes. The between-mycotype differences in the relative abundance compared to mycotype A were tested by the Wilcoxon rank-sum test. The relationships between the relative abundance and the outcomes (PPV use and hospital length of stay [LOS] of ≥2 days) were examined by using the Spearman rank correlation test. No microbial functions had a significant correlation with the outcomes (false discovery rate [FDR] ≥0.05). Abbreviations: *E. coli*, *Escherichia coli*; PPV, positive pressure ventilation.

The mycotypes showed similar profiles in bacterial composition and microbial functions (Fig. 4 and Supplementary Figure S6). Compared with mycotype A, mycotypes B, C, or D showed a similar profile in bacterial composition and had few species with significant difference (e.g., a lower abundance of *Cutibacterium acnes* in Mycotype D, FDR <0.05; Supplementary Figure S6). Additionally, mycotype C or D had no significantly different microbial functions (FDR ≥0.05), although mycotype B had major microbial functions with significantly different abundance (e.g., higher abundances of gondoate biosynthesis [anaerobic] and superpathway of fatty acid biosynthesis initiation [*E. coli*] functions; FDR <0.05; Fig. 4). In the relationship of bacterial composition and microbial functions with the outcomes, only *Moraxella catarrhalis* abundance was inversely correlated with LOS of ≥2 days (Spearman’s rho, −0.20; FDR = 0.017; Fig. 4 and Supplementary Figure S6).

### Mycotypes had distinct biological characteristics in host response in the airway

**Fig. 5:**
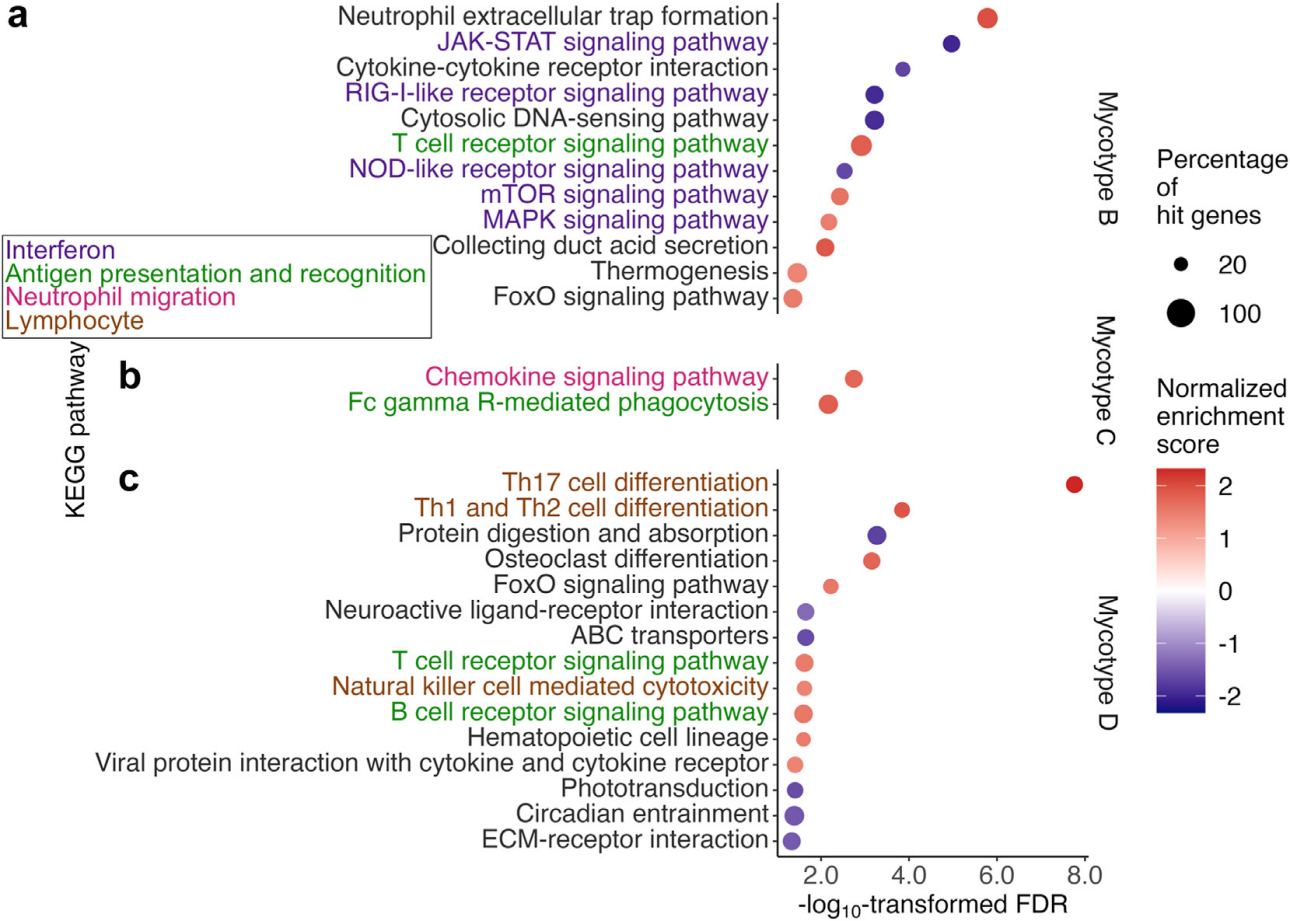
**Between-mycotype differences (A vs. B, C, and D) in nasopharyngeal host transcriptome pathways (Kyoto Encyclopedia of Genes and Genomes) among infants hospitalized for bronchiolitis.** To examine the difference in the host response in the airway between the mycotypes (A [the reference] vs. B, C, and D), the gene set enrichment analysis based on Kyoto Encyclopedia of Genes and Genomes (KEGG) is applied to the nasopharyngeal host transcriptome data. Of the pathways in Environmental Information Processing and Organismal Systems, pathways with false discovery rate (FDR) <0.05 were selected for (a) mycotype A vs. B, (b) A vs. C, and (c) A vs. D. Abbreviations: ABC, adenosine triphosphate binding cassette; ECM, extracellular matrix; Fc gamma R, Fc gamma receptor; FoxO, forkhead box O; JAK-STAT, Janus kinase-signal transducer and activator of transcription; MAPK, mitogen-activated protein kinases; mTOR, mammalian target of rapamycin; NOD, nucleotide oligomerization domain; RIG, retinoic acid-inducible gene.

The mycotypes of infant bronchiolitis had distinct biological characteristics in host airway response. Compared with mycotype A, mycotypes B, C, and D had different gene expression signatures (Supplementary Figure S7) and dysregulated pathways (FDR <0.05; Fig. 5 and Supplementary Figure S8). Of the identified pathways, mycotype C (fungus^*M.globosa*^virus^RSV/RV^) with the lowest risk of PPV use had specifically dysregulated antigen presentation and recognition (e.g., Fc γ receptor-mediated phagocytosis pathway) and neutrophil migration (e.g., chemokine signaling pathway and neutrophil migration) pathways. Additionally, mycotype D (*fungus*^not-detected^virus^RSV/RV^) had dysregulated lymphocyte pathways (e.g., T_H_17 cell differentiation).

### Sensitivity analysis

The consistency in the associations of mycotypes with the risks of PPV use was confirmed in different models. For example, compared with mycotype A, mycotype C had a lower risk of PPV use in logistic regression models with log*F*-type penalized method (adjOR, 0.20; 95% CI, 0.02–0.84; p = 0.065; Supplementary Figure S9) and modified Poisson regression models (adjusted risk ratio, 0.16; 95% CI, 0.02–1.20; p = 0.075; Supplementary Figure S10). In the analysis excluding infants with a previous history of breathing problems, the associations of mycotypes with disease severity remained consistent, albeit with limited statistical power (n = 314). For example, compared with mycotype A, mycotype C had a lower risk of PPV use (7% vs. 1%; adjOR, 0.26; 95% CI, 0.03–1.14; p = 0.079; Supplementary Figure S11). In mycotyping with three- and five-class models, the alluvial plot (Supplementary Figure S12) demonstrates a consistency of the original mycotypes (mycotypes A–D) across the different number-class chosen. For example, in a five-class model, mycotype 1, 3, or 4 had 100% concordance with the original mycotype A, B, or C, respectively (Supplementary Figure S12 and Supplementary Table S5). Compared to mycotype 1, mycotype 4—which is concordant with mycotypes C (Supplementary Figure S13)—had a lower risk of PPV use (6% vs. 1%; adjOR, 0.27; 95% CI, 0.03–1.16; p = 0.083; Supplementary Figure S14).

## Discussion

By integrating fungus and virus data from a multicentre prospective cohort of infants hospitalized for bronchiolitis, we identified four clinically distinct mycotypes that have differential severity and biological significance. For example, compared with the reference mycotype A (fungus^*M.restricta*^virus^RSV/RV^), infants with mycotype C (fungus^*M.globosa*^virus^RSV/RV^) had a significantly lower risk of PPV use. Additionally, while having similar bacterial composition and microbial functions, these infants with mycotype C had distinct host airway responses (e.g., Fc γ receptor-mediated phagocytosis pathway and chemokine signaling pathway). The sensitivity analysis revealed the robustness of our findings. The current investigation identified distinct fungus-virus subtypes of infant bronchiolitis and their relationship with the different bronchiolitis severity.

Bronchiolitis has conventionally been considered a single disease with similar pathobiological mechanisms.^5^ However, recent research has suggested that bronchiolitis is a syndrome with a high degree of microbial heterogeneity.^6^^,^^8^^,^^9^^,^^11^ The observed mycotypes are in agreement with studies that have evaluated the heterogeneity in terms of respiratory viruses, bacteria, and fungi. For example, a study in young children (age <2 years) hospitalized for bronchiolitis has reported that an RSV-RV coinfection subtype had a longer hospital length-of-stay.^6^ Additional studies of children (<2 years) hospitalized for bronchiolitis have also reported a similar virus-derived subtype-severity relationship.^8^^,^^9^ Furthermore, a study of infants (<1 year) hospitalized for bronchiolitis has reported the association of a *Haemophilus*-dominant subtype with more severe illness.^10^ Within the sparse literature on airway fungi, a birth cohort study using nasal fungus data has reported that, in 121 neonates at age one week, their fungal profiles showed a high inter-individual variation.^11^ The current study builds on these earlier reports and extends them by identifying distinct mycotypes with differential risks of PPV use and their biological significance by integrating the nasopharyngeal metatranscriptome and host transcriptome data. A better understanding of mycotypes may inform potential prophylactic and therapeutic strategies (e.g., modulating host immune response by altering airway fungal profile) against bronchiolitis in infants.

Although the exact mechanisms underlying the observed mycotypes warrant further investigation, the current study found that mycotypes A, C, and D—despite similar respiratory viral and airway bacterial profiles—had distinct disease severity risks. Indeed, compared to mycotype A (fungus^*M.restricta*^virus^RSV/RV^), mycotype C (fungus^*M.globosa*^virus^RSV/RV^, with the highest proportion of lifetime or recent corticosteroid use) had a lower risk of PPV use. Consistently, growing evidence suggests the role of *Malassezia* species—the most abundant fungal species in the upper airway of infants^12^—in the systemic and local (skin) host response and the presence or severity of diseases. For example, studies have shown that different *Malassezia* species induced distinct cytokine profiles (e.g., greater interleukin-10 induction, an anti-inflammatory cytokine, by *M. globosa* than by *M. restricta*) in keratinocytes^51^ and peripheral CD4^+^ T cells^19^ from healthy donors. Additionally, the literature has revealed a higher *M. globosa* abundance in the skin of healthy subjects than of subjects with atopic dermatitis.^52^ Another study of infants with RSV infection, with a limited sample size (n = 43), has also reported that infants with milder illness had a higher *M. globosa* abundance in their nasal cavity.^12^ Furthermore, recent research also suggests the relationship among corticosteroid use, airway fungi, and bronchiolitis severity. For example, a study in adults with asthma or allergic bronchopulmonary aspergillosis has reported that those with chronic inhaled and/or systemic corticosteroid use had a higher total fungal or *Aspergillus Fumigatus* genomic load in the bronchoalveolar lavage.^41^ In contrast, a meta-analysis study among children with bronchiolitis (<2 years) has reported no significant relationships of short-term inhaled or systemic corticosteroid use with admissions risk or LOS.^53^ These data collectively suggest a protective role of *Malassezia* species in acute respiratory infection.

We also observed that these infants with mycotype C (fungus^*M.globosa*^virus^RSV/RV^) had dysregulated antigen presentation and recognition (e.g., Fc γ receptor-mediated phagocytosis pathway) and neutrophil migration (e.g., chemokine signaling pathway and neutrophil migration) pathways. Consistently, recent studies have shown the relationship among *Malassezia* species, antigen presentation and recognition, neutrophil migration, and severe illness in respiratory infection. For example, murine model studies have shown that *Malassezia* species were recognized by C-type lectin receptors (e.g., Mincle) that couple to the Fc receptor γ chain in activated antigen presenting cells (e.g., macrophages and dendritic cells) and transduce the signals to produce neutrophil attracting chemokines (e.g., CXCL1, CXCL2).^54^, ^55^, ^56^ Murine model studies have also reported that *M. pachydermatis*^57^ or protease derived from *M. furfur*^58^ increased a chemokine gene expression (e.g., *Cxcl1*^57^ or *Cxcl5*^58^) and neutrophil cell count in their barrier-compromised skin. Regarding the relationship between neutrophil migration and RSV severity, a study in infants with RSV infection has shown that those without dyspnoea had a lower urinary trypsin inhibitor level—a marker of neutrophil activation—than those with dyspnoea.^59^ Another study has shown that, among healthy adults administered RSV, those who developed no symptoms had a lower protein level of neutrophil-associated mediators (e.g., myeloperoxidase) before the administration than those who developed symptoms.^60^ Taken together, these studies indicate the role of airway *Malassezia* species in antigen presentation and recognition, neutrophil migration, and disease severity.

Our data also showed that mycotype D (fungus^not-detected^virus^RSV/RV^) has dysregulated lymphocyte pathways (e.g., T_H_17 cell differentiation). Consistently, recent evidence also suggests the relationship between *Malassezia* species and T_H_17 immunity. For example, research has revealed that, among *M. restricta*, *M. globosa*, and *Malassezia furfur*, *M. globosa* most strongly induced IL-17A positive CD4^+^ T cells in peripheral blood mononuclear cells from healthy donors.^19^ A murine model study has also reported that *Malassezia pachydermatis* promoted inflammation in their barrier-disrupted skin by increasing IL-17A production by CD4^+^ T cells^57^. Regardless of the complexity of these potential mechanisms, the current study has demonstrated distinct bronchiolitis mycotypes that have unique host airway responses and differential disease severity risks. Our data should advance research into developing prophylactic and treatment strategies for infant bronchiolitis.

The current study has several potential limitations. First, the study did not have non-bronchiolitis “controls”. However, this study did not aim to elicit infant mycotypes related to the development of bronchiolitis (i.e., the cases vs. the controls) but to examine bronchiolitis mycotypes and their disease severity risks. Secondary, the exact clinical presentations at the timing of PPV use were not available, and PPV use was determined at the discretion of the treating physician. Therefore, among infants who underwent PPV, the bronchiolitis severity may have differed. However, PPV use should be a more specific outcome for bronchiolitis severity than other variables (e.g., LOS or admission to intensive care unit). Third, although bronchiolitis involves microorganisms and inflammation in both upper and lower airways, the current study relied on upper airway data. Nonetheless, research on children has shown that upper airway data reliably represents the microbiome^61^ and transcriptome^62^ profiles in the lower airway, which is more invasive and difficult to collect in young infants. Fourth, our mycotypes based on RNA-seq data (i.e., shotgun sequencing)—not by different methods, such as marker gene sequencing (e.g., nuclear ribosomal internal transcribed spacer region)—of nasopharyngeal specimens that may have low biomass. Fifth, our inferences may be biased by the sparse outcome data and the limited number of adjusted confounders. Regarding the sparse outcome data, we confirmed the consistency of inference among different models (e.g., logistic regression models with Firth’s penalized method or log*F-*type penalized method and modified Poisson regression models). Regarding the limited number of adjusted confounders, in the association of mycotype C with the risk, while the low E-value for the upper 95% confidence limit of odds ratio (1.47) would suggests the statistical uncertainty of the model (i.e., the association could be moved to include the null by a set of not substantial unmeasured confounders), the high E-value for the point estimate (8.86) would support the robustness (i.e., the association would be explained away only by a set of substantial unmeasured confounders). Sixth, our inferences may also be biased due to the relationship between the timing of specimen collections, treatments, and PPV use. However, previous research has shown that, *in vitro*, *M. restricta* and *M. globosa* grew quite slowly, taking longer than a month to develop sufficient biomass.^63^^,^^64^ Seventh, while our findings are clinically and biologically plausible, they warrant further validation in an independent cohort and mechanistic study. Lastly, although the study sample consisted of a geographically and racially/ethnically diverse cohort, our inferences should be generalized cautiously to populations other than infants hospitalized for bronchiolitis. Nevertheless, our findings are highly relevant to >110,000 hospitalized infants annually in the U.S.—a population with a substantial morbidity burden.^2^

By applying unsupervised clustering approaches to the fungus and virus data from a prospective multicenter cohort study of infants hospitalized for bronchiolitis, we identified four clinically meaningful mycotypes. Specifically, the mycotype characterized by the highest *M. globosa* relative abundance and RSV and RV infection had the lowest bronchiolitis severity. Additionally, by using the nasopharyngeal metatranscriptome and host transcriptome data, these mycotypes revealed distinct biological characteristics (e.g., dysregulated antigen presentation and recognition and neutrophil migration pathways in transcriptome data) during bronchiolitis. Our findings should facilitate further research into interplay between fungi, respiratory viruses, and host airway response in bronchiolitis pathobiology. Furthermore, these findings should offer an evidence-base for developing potential prophylactic and therapeutic strategies (e.g., modulating host immune response by altering airway fungal profile) in this large patient population with a high morbidity burden.

## Contributors

Dr. Shibata carried out the main statistical analysis, drafted the initial manuscript, and approved the final manuscript as submitted. Drs. Zhu, Kyo, and Ooka verified the underlying data, reviewed and revised the initial manuscript, and approved the final manuscript as submitted. Drs. Freishtat and Mansbach collected the study data, reviewed and revised the initial manuscript, and approved the final manuscript as submitted. Dr. Pérez-Losada carried out the bioinformatic analyses of the genomic data, reviewed and revised the initial manuscript, and approved the final manuscript as submitted. Drs. Hasegawa and Camargo conceptualized the study, obtained funding, supervised the statistical analysis, reviewed and revised the initial manuscript, and approved the final manuscript as submitted.

## Data sharing statement

The RNA-seq data that support the findings of this study are available on the NIH/NIAID ImmPort (https://www.immport.org/shared/study/SDY1883), with the accession number SDY1883, upon reasonable request from the researchers, whose research investigates severe bronchiolitis, recurrent wheezing, asthma, and related concepts. The data are not publicly available to be compliant with the informed consent forms of MARC-35 study and the genomic data sharing plan.

## Declaration of interests

All authors have no conflicts of interest relevant to this article to disclose.

## Appendix A Supplementary data

Supplementary material

SampleCode
