# Supplementary material for: Nasopharyngeal fungal subtypes of infant bronchiolitis and disease severity risk
Source: eBioMedicine. 2023 Aug 1;95:104742. doi: 10.1016/j.ebiom.2023.104742 (PMC10415709; doi:10.1016/j.ebiom.2023.104742)
Supplement: Supplementary material [file mmc1.docx]

SUPPLEMENTARY MATERIAL

**Nasopharyngeal fungal subtypes of infant bronchiolitis and disease severity risk**

Ryohei Shibata, MD, PhD; Zhaozhong Zhu, ScD; Michihito Kyo, MD, PhD; Tadao Ooka, MD, PhD; Robert J. Freishtat, MD, MPH; Jonathan M. Mansbach, MD, MPH; Marcos Pérez-Losada, PhD; Carlos A. Camargo Jr., MD, DrPH; and Kohei Hasegawa, MD, MPH, PhD

**Table of Contents:**

[Supplementary Methods 2](#_Toc138949360)

[Supplementary References (the style will be formatted before submission) 5](#_Toc138949361)

[Table S1. Principal investigators at the 17 participating sites in MARC-35 7](#_Toc138949362)

[Table S2. Comparisons between analytic and non-analytic cohorts in MARC-35 8](#_Toc138949363)

[Table S3. Comparisons between male and female sexes 10](#_Toc138949364)

[Table S4. Relative abundance and carriage of the 10 most abundant nasopharyngeal fungal species among infants hospitalized for bronchiolitis 12](#_Toc138949365)

[Table S5. Baseline patient characteristics and clinical course of infants hospitalized for bronchiolitis using five mycotypes in the sensitivity analysis 13](#_Toc138949366)

[Figure S1. Path plot to identify an optimal number of the fungus clusters 16](#_Toc138949367)

[Figure S2. Consensus matrices, cumulative distribution function plot, and cluster consensus plots to identify an optimal number of the virus clusters 17](#_Toc138949368)

[Figure S3. Consensus matrices, cumulative distribution function plot, and cluster consensus plots to identify an optimal number of the fungus-virus clusters 18](#_Toc138949369)

[Figure S4. Causal directed acyclic graph 19](#_Toc138949370)

[Figure S5. Association of mycotypes of infant bronchiolitis with risk of length of hospital stay of ≥2 days 20](#_Toc138949371)

[Figure S6. Between-mycotype difference in Shannon index and relative abundance of the 25 most abundant nasopharyngeal bacterial species among infants hospitalized for bronchiolitis 21](#_Toc138949372)

[Figure S7. Differential host gene expression analysis between mycotypes in infants hospitalized for bronchiolitis 22](#_Toc138949373)

[Figure S8. Between-mycotype differences in nasopharyngeal host transcriptome pathways (Gene Ontology) among infants hospitalized for bronchiolitis 23](#_Toc138949374)

[Figure S9. Sensitivity analysis: Association of mycotypes of infant bronchiolitis with risk of positive pressure ventilation use by logistic regression models with log*F*-type penalized method 24](#_Toc138949375)

[Figure S10. Sensitivity analysis: Association of mycotypes of infant bronchiolitis with risk of positive pressure ventilation use by modified Poisson regression models 25](#_Toc138949376)

[Figure S11. Sensitivity analysis: Association of mycotypes of infant bronchiolitis with risk of positive pressure ventilation use, limiting to infants without a previous breathing problem 26](#_Toc138949377)

[Figure S12. Alluvial plot to examine consistencies across different numbers of mycotypes 27](#_Toc138949378)

[Figure S13. Sensitivity analysis: Clinical, virus, and fungus characteristics of infants hospitalized for bronchiolitis, according to mycotypes, by using five mycotypes 28](#_Toc138949379)

[Figure S14. Sensitivity analysis: Association of mycotypes of infant bronchiolitis with risk of positive pressure ventilation use, using five mycotypes 29](#_Toc138949380)

# Supplementary Methods

**Study design, setting, and participants**

We analysed data from an ongoing, multicentre, prospective cohort study of infants (age < 1 year) hospitalized for bronchiolitis. This study, called the 35th Multicenter Airway Research Collaboration (MARC-35) [1–3], is coordinated by the Emergency Medicine Network (EMNet) [4], a research collaboration of 247 participating hospitals. During designing this study, sex was not taken into account. Using a standardized protocol [1–3], the investigators at 17 sites across 14 U.S. states (**Table S1**) enrolled infants hospitalized with an attending physician diagnosis of bronchiolitis during one of the consecutive bronchiolitis seasons from November 1 to April 30 (2011–2014). Bronchiolitis was defined by the American Academy of Pediatrics (AAP) guidelines—acute respiratory illness with some combination of rhinitis, cough, tachypnoea, wheezing, crackles, and retractions, regardless of previous breathing problem episodes [5]. We excluded infants with a known heart-lung disease, immunodeficiency, immunosuppression, or gestational age <32 weeks.

**Ethics**

This study involves human participants and was approved by the Human Research Committee at Massachusetts General Hospital (protocol 2017P001861). Participants gave informed consent to participate in the study before taking part.

**Data collection**

Clinical data (patients’ demographic characteristics, medical history, environmental, and family, and details of the acute illness) were collected via structured interview and chart reviews [1]. Sex data was collected through reports provided by the parent or guardian. All data were reviewed at the EMNet Coordinating Center at Massachusetts General Hospital (Boston, MA, U.S.A.), and site investigators were queried about missing data and discrepancies identified by manual data checks.

Nasopharyngeal specimens were collected by trained site investigators within 24 hours of hospitalization and immediately placed on ice and then stored at −80°C using standard protocols [1,6]. All sites used the same collection equipment (Medline Industries, Mundelein, IL, U.S.A.). The frozen specimens were shipped in batches to Baylor College of Medicine (Houston, TX, U.S.A.) where they were tested for 17 respiratory viruses (including respiratory syncytial virus [RSV] and rhinovirus [RV]) using real-time polymerase chain reaction (RT-PCR) assays [1,6,7]. For RV detection, complementary DNA was generated using virus-specific primers for RV, and singleplex RT-PCR was used. The details of the RV primers and probes have been described elsewhere [8]. Next, to identify the RV species (A and C), RV-positive samples were partially sequenced to determine the RV species and type at the University of Wisconsin Madison (Madison, WI, U.S.A.) [9]. The frozen specimens were also shipped to the University of Maryland (Baltimore, MD, U.S.A.) for RNA sequencing (RNA-seq).

**Nasopharyngeal total RNA extraction and RNA-seq**

Total RNA was isolated from 398 randomly-selected nasopharyngeal specimens using Trizol LS reagent (ThermoFisher Scientific, Waltham, MA, U.S.A.) in combination with the Direct-zol RNA Miniprep Kit (Zymo Research, Irvine, CA, U.S.A.). RNA quantity was measured with the Qubit 2.0 fluorometer (ThermoFisher Scientific). Its quality was assessed with the Agilent Bioanalyzer 2100 (Agilent, Palo Alto, CA, U.S.A.) using the RNA 6000 Nano kit. Total RNA underwent DNase treatment using the TURBO DNA-free™ Kit (ThermoFisher Scientific) and rRNA reduction for both human and bacterial rRNA using NEBNext rRNA Depletion Kits (New England Biolabs, Ipswich, MA, U.S.). RNA was prepared for sequencing using the NEBNext Ultra II Directional RNA Library Prep Kit (New England Biolabs) and sequenced on a NovaSeq6000 (Illumina, San Diego, CA, U.S.A.) using an S4 100 bp PE Flowcell (Illumina). All RNA-seq samples had sufficient sequence depth (mean, 48,014,679 pair-end reads/sample) to obtain a high degree of sequence coverage.

**Nasopharyngeal metatranscriptome profiling**

Raw sequence reads were filtered and trimmed for adapters and contaminant reads (e.g., reads mapping to human genes and transcripts and bacterial rRNA) using KneadData v0.10.0 [10]. For nasopharyngeal metatranscriptome profiling, we first inferred fungal taxonomy by EukDetect [11] using default settings. In summary, the filtered and trimmed reads were aligned against the EukDetect marker database built on January 22, 2022 (available from: <https://figshare.com/articles/dataset/Eukdetect_database/12670856/8>) and underwent quality filtering. Next, reads per kilobase of sequence (RPKS; i.e., absolute abundance) and the relative abundance (EukFrac) were calculated. Second, we inferred bacterial taxonomy by MetaPhlAn 3.0 [12] using the ChocoPhlAn database v30 [12] and obtained 5,603,386,939 merged sequences. Third, we conducted microbial functional profiling. The filtered and trimmed reads were aligned to UniRef90 by DIAMOND [13], annotated into MetaCyc metabolic pathways [14] by HUMAnN 3.0 [12], and mapped against this database to quantify pathway presence and abundance. Finally, we normalized bacterial composition and microbial function raw values (i.e., reads per kilobase) to relative abundance by total-sum scaling.

**Nasopharyngeal host transcriptome profiling**

Raw sequence reads were trimmed and filtered for adapters and contaminants using the *k*-mers strategy in bbduk [15] and default settings (i.e., minlength=14, minavgquality=20, maxns=0). We estimated transcript abundances from the filtered and trimmed reads by Salmon 1.9.0 [16] using the human transcriptome (hg38) and the mapping-based mode. We first generated a decoy-aware transcriptome and then quantified the reads using Salmon’s default settings and the following flags: –validateMappings, – recoverOrphans, –seqBias, and –gcBias. Salmon is fast and accurate, corrects for potential changes in gene length across samples (e.g., from differential isoform usage), and has higher sensitivity at the same false discovery rate (FDR) in differential expression gene analysis. Next, by calculating log10-transformed total read count per specimen, we filtered the 14 mRNA data with the low read count defined as less than the mean minus 2.5 standard deviations. Lastly, we normalize the read count by the R *DESeq2* package [17] using default settings.

**Outcome measure**

The clinical outcome of interest was acute severity of bronchiolitis. Specifically, the primary outcome was the use of positive pressure ventilation (PPV), defined as the use of continuous positive airway pressure ventilation and/or mechanical ventilation during the index hospitalization [2]. The secondary outcome was a hospital length of stay (LOS) of ≥2 days, defined using the median LOS of 2 days.

**Statistical analysis**

The objectives of the current study are 1) to identify mycotypes among infants hospitalized for bronchiolitis, 2) to examine their associations with the risk of PPV use, and 3) to determine their biological characteristics with the use of the nasopharyngeal metatranscriptome and host transcriptome data. The analytic workflow is summarized in **Figure 1**.

First, we identified mutually exclusive clusters for each of the fungus and virus datasets of MARC-35 infants collected at the index hospitalization. For the fungus data where at least one fungal species was detected, we used the relative abundance data of nasopharyngeal fungal species and derived the clusters by a Dirichlet multinomial mixtures (DMM) model using the R *DirichletMultinomial* package [18]. This model describes microbial composition data by Dirichlet and multinomial distributions and determines clusters by deeming the Dirichlet distribution as a mixture derived from different subtypes. To choose an optimal number of these clusters, we used a Laplace approximation to the negative log model evidence. Additionally, we grouped one cluster by the fungus data without detectable fungi, added this cluster to the clusters based on a DMM model, and generated the fungus clusters. For the virus data, we used RSV (binary), RV species A and C (binary), and their genomic load (count) represented by cycle threshold (Ct) values based on their importance in bronchiolitis pathobiology and severity [6]. When the virus was not detected, we imputed the Ct values with 40. Then, we computed a Gower distance using the R *StatMatch* package [19] and derived the virus clusters by using a consensus clustering algorithm with partitioning around medoids (PAM) method using the R *ConsensusClusterPlus* package [20,21]. To choose an optimal number of the virus clusters, we used a combination of separations of the consensus matrix, consensus cumulative distribution function (CDF), and cluster consensus value, rather than using the indices separately, in addition to the cluster size and clinical plausibility [6,22,23].

Second, we combined the fungus and virus clusters to derive a fused matrix, computed a Gower distance, and identified mutually exclusive four mycotypes by using a consensus clustering algorithm with PAM method.

Third, to interpret the clinical and virus characteristics of the four mycotypes, we compared the baseline clinical and virus characteristics by using the chi-square and Kruskal-Wallis tests as appropriate. Additionally, we developed chord diagrams on the relationship of the mycotypes with major clinical and virus variables using the R *circlize* package [24]. Additionally, to interpret the fungus characteristics of the four mycotypes, we developed bee swarm plots by the Shannon index using the R *vegan* package [25] as well as the relative and absolute abundance of the two most abundant nasopharyngeal fungal species.

Fourth, to determine the association of the mycotypes (the exposure) with the risk of PPV use and a LOS ≥2 days (the outcomes), we first constructed a directed acyclic graph to represent our causal structure hypothesis, along with potential confounders (i.e., age and use of systemic corticosteroids for breathing problems that caused the index hospitalization). The potential confounders were selected based on clinical plausibility, *a priori* knowledge [26–29] and the number of patients with the outcome. Next, we constructed unadjusted and multivariable logistic regression models. Regarding the association with the risk of PPV use, the models were corrected by Firth’s penalized method to reduce a bias of maximum likelihood estimate for a low proportion of the outcome [30]. In the multivariable models, we adjusted for the potential confounders.

Fifth, we examined the difference in biological characteristics in nasopharyngeal bacterial composition and microbial functions between the derived mycotypes by using the metatranscriptome data. To examine the between-mycotype difference, we created heatmaps by the Shannon index and relative abundance of the 25 most abundant bacterial species and microbial functions. The between-mycotype differences were tested by the Wilcoxon rank-sum test. Additionally, to examine the relationships of the bacterial composition and microbial functions with the outcomes, by using the relative abundance and the binary outcome data, we summarized Spearman’s rho in the heatmaps. Lastly, we examined the between-mycotype difference in biological characteristics in host airway response by using the host transcriptome data. We first conducted differential expression gene analysis with adjustment for potential confounders (age and use of systemic corticosteroids for breathing problems that caused the index hospitalization) between the mycotypes by using the R *DESeq2* package [17]. Next, by using the rank gene list with Wald statistic (i.e., log_2_ fold change divided by standard error) in the differential expression gene analysis, we conducted Gene Set Enrichment Analysis (GSEA) [31,32] based on Environmental Information Processing and Organismal Systems in Kyoto Encyclopedia of Genes and Genomes PATHWAY [33] and descendants of GO:0002376 immune system process in Biological Processes of Gene Ontology by the R *clusterProfiler* package (FDR <0.05) [34,35].

In the sensitivity analysis, we first computed E-values to determine the robustness of causal inference to potential unmeasured confounding by using the R *EValue* package [36]. The E-value represents the minimum magnitude of association that a set of unmeasured confounders would need to have in order to fully explain the association of interest, conditional on the covariates. For example, an E-value of 2.0 means that the odds ratio for the association of unmeasured confounders with both the exposure and outcome would have to be ≥2.0 to explain away the observed exposure-outcome association. Next, we confirmed the mycotype-outcome associations by constructing different models. For example, considering that Firth’s penalized method could lead to artifacts (e.g., the estimates fall outside the range of the prior median and the maximum likelihood estimate), we constructed logistic regression models with the log*F*-type penalized method by using the R *logistlogF* package [39,40]. We also estimated risk ratios by constructing modified Poisson regression models [41] (i.e., Poisson regression models followed by sandwich error estimation using the R *lmtest* package [42]). Additionally, we examined the mycotype-outcome associations after excluding infants with a previous history of breathing problem. Lastly, we also examined the robustness of the mycotype-outcome associations by repeating the analysis using a different number of mycotypes.

Analysis used R version 4.1.3 (R Foundation, Vienna, Austria). All P-values were two-tailed, with *p* <0.05 considered statistically significant. We computed the Benjamin-Hochberg FDR that allows for the interpretation of statistical significance in the context of multiple hypothesis testing [43], with FDR <0.05 considered statistically significant.

# Supplementary References (the style will be formatted before submission)

[1] Hasegawa K, Mansbach JM, Ajami NJ, Espinola JA, Henke DM, Petrosino JF, et al. Association of nasopharyngeal microbiota profiles with bronchiolitis severity in infants hospitalised for bronchiolitis. Eur Respir J 2016;48:1329–39.

[2] Stewart CJ, Mansbach JM, Wong MC, Ajami NJ, Petrosino JF, Camargo CA Jr, et al. Associations of Nasopharyngeal Metabolome and Microbiome with Severity among Infants with Bronchiolitis. A Multiomic Analysis. Am J Respir Crit Care Med 2017;196:882–91.

[3] Hasegawa K, Mansbach JM, Ajami NJ, Petrosino JF, Freishtat RJ, Teach SJ, et al. Serum cathelicidin, nasopharyngeal microbiota, and disease severity among infants hospitalized with bronchiolitis. J Allergy Clin Immunol 2017;139:1383-1386.e6.

[4] Emergency Medicine Network n.d.

[5] Ralston SL, Lieberthal AS, Meissner HC. Ralston SL, Lieberthal AS, Meissner HC, et al. Clinical Practice Guideline: The Diagnosis, Management, and Prevention of Bronchiolitis. Pediatrics. 2014;134(5):e1474-e1502. Pediatrics 2015;136:782.

[6] Hasegawa K, Jartti T, Mansbach JM, Laham FR, Jewell AM, Espinola JA, et al. Respiratory syncytial virus genomic load and disease severity among children hospitalized with bronchiolitis: multicenter cohort studies in the United States and Finland. J Infect Dis 2015;211:1550–9.

[7] Mansbach JM, Piedra PA, Stevenson MD, Sullivan AF, Forgey TF, Clark S, et al. Prospective multicenter study of children with bronchiolitis requiring mechanical ventilation. Pediatrics 2012;130:e492-500.

[8] Lu X, Holloway B, Dare RK, Kuypers J, Yagi S, Williams JV, et al. Real-time reverse transcription-PCR assay for comprehensive detection of human rhinoviruses. J Clin Microbiol 2008;46:533–9.

[9] Bochkov YA, Grindle K, Vang F, Evans MD, Gern JE. Improved molecular typing assay for rhinovirus species A, B, and C. J Clin Microbiol 2014;52:2461–71.

[10] McIver LJ, Abu-Ali G, Franzosa EA, Schwager R, Morgan XC, Waldron L, et al. bioBakery: a meta’omic analysis environment. Bioinformatics 2018;34:1235–7.

[11] Lind AL, Pollard KS. Accurate and sensitive detection of microbial eukaryotes from whole metagenome shotgun sequencing. Microbiome 2021;9:58.

[12] Beghini F, McIver LJ, Blanco-Míguez A, Dubois L, Asnicar F, Maharjan S, et al. Integrating taxonomic, functional, and strain-level profiling of diverse microbial communities with bioBakery 3. Elife 2021;10. https://doi.org/10.7554/eLife.65088.

[13] Buchfink B, Xie C, Huson DH. Fast and sensitive protein alignment using DIAMOND. Nat Methods 2014;12:59–60.

[14] Caspi R, Altman T, Billington R, Dreher K, Foerster H, Fulcher CA, et al. The MetaCyc database of metabolic pathways and enzymes and the BioCyc collection of Pathway/Genome Databases. Nucleic Acids Res 2014;42:D459-71.

[15] Bushnell B. BBMap: a fast, accurate, splice-aware aligner. Lawrence Berkeley National Lab.(LBNL), Berkeley, CA (United States); 2014.

[16] Patro R, Duggal G, Love MI, Irizarry RA, Kingsford C. Salmon provides fast and bias-aware quantification of transcript expression. Nat Methods 2017;14:417–9.

[17] Love MI, Huber W, Anders S. Moderated estimation of fold change and dispersion for RNA-seq data with DESeq2. Genome Biol 2014;15:550.

[18] Holmes I, Harris K, Quince C. Dirichlet Multinomial Mixtures: Generative Models for Microbial Metagenomics. PLoS One 2012;7:e30126.

[19] D’Orazio, D’Orazio. Package “StatMatch.” Available Online at One of the Mirror 2022.

[20] Wilkerson MD, Hayes DN. ConsensusClusterPlus: a class discovery tool with confidence assessments and item tracking. Bioinformatics 2010;26:1572–3.

[21] Monti S, Tamayo P, Mesirov J, Golub T. Consensus Clustering: A Resampling-Based Method for Class Discovery and Visualization of Gene Expression Microarray Data. Mach Learn 2003;52:91–118.

[22] Mansbach JM, Piedra PA, Teach SJ, Sullivan AF, Forgey T, Clark S, et al. Prospective multicenter study of viral etiology and hospital length of stay in children with severe bronchiolitis. Arch Pediatr Adolesc Med 2012;166:700–6.

[23] Dumas O, Mansbach JM, Jartti T, Hasegawa K, Sullivan AF, Piedra PA, et al. A clustering approach to identify severe bronchiolitis profiles in children. Thorax 2016;71:712–8.

[24] Gu Z, Gu L, Eils R, Schlesner M, Brors B. circlize Implements and enhances circular visualization in R. Bioinformatics 2014;30:2811–2.

[25] Oksanen J, Blanchet FG, Kindt R, Legendre P, Minchin PR, O’hara RB, et al. Package ‘vegan.’ Community Ecology Package, Version 2013;2:1–295.

[26] Fraczek MG, Chishimba L, Niven RM, Bromley M, Simpson A, Smyth L, et al. Corticosteroid treatment is associated with increased filamentous fungal burden in allergic fungal disease. J Allergy Clin Immunol 2018;142:407–14.

[27] Havdal LB, Bøås H, Bekkevold T, Bakken Kran A-M, Rojahn AE, Størdal K, et al. Risk factors associated with severe disease in respiratory syncytial virus infected children under 5 years of age. Front Pediatr 2022;10:1004739.

[28] Rajagopala SV, Bakhoum NG, Pakala SB, Shilts MH, Rosas-Salazar C, Mai A, et al. Metatranscriptomics to characterize respiratory virome, microbiome, and host response directly from clinical samples. Cell Rep Methods 2021;1. https://doi.org/10.1016/j.crmeth.2021.100091.

[29] Pattaroni C, Macowan M, Chatzis R, Daunt C, Custovic A, Shields MD, et al. Early life inter-kingdom interactions shape the immunological environment of the airways. Microbiome 2022;10:34.

[30] Firth D. Bias reduction of maximum likelihood estimates. Biometrika 1993;80:27–38.

[31] Subramanian A, Tamayo P, Mootha VK, Mukherjee S, Ebert BL, Gillette MA, et al. Gene set enrichment analysis: a knowledge-based approach for interpreting genome-wide expression profiles. Proc Natl Acad Sci U S A 2005;102:15545–50.

[32] Mootha VK, Lindgren CM, Eriksson K-F, Subramanian A, Sihag S, Lehar J, et al. PGC-1alpha-responsive genes involved in oxidative phosphorylation are coordinately downregulated in human diabetes. Nat Genet 2003;34:267–73.

[33] Kanehisa M, Furumichi M, Sato Y, Kawashima M, Ishiguro-Watanabe M. KEGG for taxonomy-based analysis of pathways and genomes. Nucleic Acids Res 2023;51:D587–92.

[34] Yu G, Wang L-G, Han Y, He Q-Y. clusterProfiler: an R package for comparing biological themes among gene clusters. OMICS 2012;16:284–7.

[35] Wu T, Hu E, Xu S, Chen M, Guo P, Dai Z, et al. clusterProfiler 4.0: A universal enrichment tool for interpreting omics data. Innovation (Camb) 2021;2:100141.

[36] Mathur MB, Smith LH, Ding P, VanderWeele TJ, Mathur MMB. Package ‘EValue.’ Package ‘EValue’ 2021.

[37] Greenland S, Mansournia MA. Penalization, bias reduction, and default priors in logistic and related categorical and survival regressions. Stat Med 2015;34:3133–43.

[38] Greenland S, Mansournia MA, Altman DG. Sparse data bias: a problem hiding in plain sight. BMJ 2016;352:i1981.

[39] logistlogF: Penalized logistic and conditional logistic regression. Github; n.d.

[40] Yu Y, Chen S, Jones SJ, Hoque R, Vishnyakova O, Brooks-Wilson A, et al. Penalized Logistic Regression Analysis for Genetic Association Studies of Binary Phenotypes. Hum Hered 2022. https://doi.org/10.1159/000525650.

[41] Zou G. A Modified Poisson Regression Approach to Prospective Studies with Binary Data. Am J Epidemiol 2004;159:702–6.

[42] Hothorn T, Zeileis A, Farebrother RW, Cummins C, Millo G, Mitchell D, et al. Package ‘lmtest.’ Testing Linear Regression Models Https://Cran r-Project Org/Web/Packages/Lmtest/Lmtest Pdf Accessed 2015;6.

[43] Benjamini Y, Hochberg Y. Controlling the false discovery rate: A practical and powerful approach to multiple testing. J R Stat Soc 1995;57:289–300.

# Table S1. Principal investigators at the 17 participating sites in MARC-35

| Amy D. Thompson, MD | Alfred I. duPont Hospital for Children, Wilmington, DE |
| --- | --- |
| Federico R. Laham, MD, MS | Arnold Palmer Hospital for Children, Orlando, FL |
| Jonathan M. Mansbach, MD, MPH | Boston Children’s Hospital, Boston, MA |
| Vincent J. Wang, MD, MHA and Susan Wu, MD | Children’s Hospital of Los Angeles, Los Angeles, CA |
| Michelle B. Dunn, MD and Jonathan M. Spergel, MD, PhD | Children’s Hospital of Philadelphia, Philadelphia, PA |
| Juan C. Celedón, MD, DrPH | Children’s Hospital of Pittsburgh, Pittsburgh, PA |
| Michael R. Gomez, MD, MS-HCA and Nancy Inhofe, MD | The Children’s Hospital at St. Francis, Tulsa, OK |
| Brian M. Pate, MD and Henry T. Puls, MD | The Children’s Mercy Hospital & Clinics, Kansas City, MO |
| Stephen J. Teach, MD, MPH | Children’s National Medical Center, Washington, D.C. |
| Richard T. Strait, MD and Stephen C. Porter, MD, MSc, MPH | Cincinnati Children’s Hospital and Medical Center, Cincinnati, OH |
| Ilana Y. Waynik, MD | Connecticut Children’s Medical Center, Hartford, CT |
| Sujit Iyer, MD | Dell Children’s Medical Center of Central Texas, Austin, TX |
| Michelle D. Stevenson, MD, MS | Norton Children’s Hospital, Louisville, KY |
| Margaret Samuels-Kalow, MD, MPhil, Wayne G. Shreffler, MD, PhD, Ari R. Cohen, MD | Massachusetts General Hospital, Boston, MA |
| Anne K. Beasley, MD and Cindy S. Bauer, MD | Phoenix Children’s Hospital, Phoenix, AZ |
| Thida Ong, MD and Markus Boos, MD, PhD | Seattle Children’s Hospital, Seattle, WA |
| Charles G. Macias, MD, MPH | Texas Children's Hospital, Houston, TX |

# Table S2. Comparisons between analytic and non-analytic cohorts in MARC-35

| **Variables** | **MARC-35**  **(n = 1,016)** | **Analytic**  **Cohort**  **(n = 398; 39%)** | **Non-analytic**  **cohort**  **(n = 618; 61%)** | **p value^*^** |
| --- | --- | --- | --- | --- |
| Demographics |  |  |  |  |
| Age, month |  |  |  | 0.28 |
| <2 | 311 (31) | 120 (30) | 191 (31) |  |
| 2–5 | 378 (37) | 139 (35) | 239 (39) |  |
| 5–11.9 | 327 (32) | 139 (35) | 188 (30) |  |
| Male sex | 610 (60) | 233 (59) | 377 (61) | 0.47 |
| Race/ethnicity |  |  |  | 0.58 |
| Non-Hispanic white | 430 (42) | 161 (40) | 269 (44) |  |
| Non-Hispanic black | 239 (24) | 91 (23) | 148 (24) |  |
| Hispanic | 308 (30) | 129 (32) | 179 (29) |  |
| Other | 39 (4) | 17 (4) | 22 (4) |  |
| C-section delivery | 348 (34) | 138 (35) | 210 (34) | 0.89 |
| Prematurity (32–36.9 weeks) | 186 (18) | 72 (18) | 114 (18) | 0.93 |
| History of eczema | 149 (15) | 61 (15) | 88 (14) | 0.65 |
| Previous breathing problems (count) |  |  |  | 0.47 |
| 0 | 810 (80) | 314 (79) | 496 (80) |  |
| 1 | 160 (16) | 62 (16) | 98 (16) |  |
| ≥2 | 46 (5) | 22 (6) | 24 (4) |  |
| Ever attended daycare | 234 (23) | 95 (24) | 139 (22) | 0.65 |
| Cigarette smoke exposure at home | 156 (15) | 60 (15) | 96 (16) | 0.86 |
| Mostly breastfed during 0-2.9 months | 423 (42) | 166 (42) | 257 (42) | 0.78 |
| Lifetime antibiotics use | 318 (31) | 126 (32) | 192 (31) | 0.89 |
| Lifetime corticosteroid use^‡^ | 147 (14) | 62 (16) | 85 (14) | 0.46 |
| Recent corticosteroid use^§^ | 98 (10) | 42 (11) | 56 (9) | 0.45 |
| Maternal smoking during pregnancy | 147 (14) | 62 (16) | 85 (14) | 0.46 |
| Clinical presentation at index hospitalization |  |  |  |  |
| Weight (kg), median (IQR) | 6.1 (4.7–7.7) | 6.2 (4.7–8.0) | 6.0 (4.7–7.6) | 0.23**^†^** |
| Respiratory rate (per minute), median (IQR) | 48 (40–60) | 50 (40–60) | 48 (40–60) | 0.33**^†^** |
| Oxygen saturation |  |  |  | 0.33 |
| <90% | 190 (9) | 37 (10) | 54 (9) |  |
| 90–93.9% | 155 (15) | 56 (14) | 99 (16) |  |
| ≥94% | 749 (74) | 294 (74) | 455 (74) |  |
| Viral testing |  |  |  |  |
| RSV | 821 (81) | 317 (80) | 504 (82) | 0.46 |
| Solo-RSV | 586 (58) | 225 (57) | 361 (58) | 0.56 |
| RSV cycle threshold values, median (IQR) | 22 (20–25) | 22 (20–25) | 22 (21–25) | 0.0058**^†^** |
| RV | 214 (21) | 86 (22) | 128 (21) | 0.75 |
| Non-RSV and non-RV | 103 (10) | 42 (11) | 61 (10) | 0.75 |
| Other pathogens^¶^ | 237 (23) | 99 (25) | 138 (22) | 0.36 |
| Clinical course |  |  |  |  |
| Received antibiotics during pre-hospitalization visit | 179 (18) | 68 (17) | 111 (18) | 0.76 |
| Received corticosteroids during pre-hospitalization visit | 90 (9) | 37 (9) | 53 (9) | 0.79 |
| Positive pressure ventilation use**^**^** | 55 (5) | 10 (5) | 45 (5) | 1.00 |
| Intensive treatment use**^††^** | 163 (16) | 26 (14) | 137 (16) | 0.55 |
| Hospital length of stay (days), median (IQR) | 2 (1–3) | 2 (1–3) | 2 (1–3) | 0.42**^†^** |
| Hospital length of stay ≥2 days | 692 (68) | 265 (67) | 427 (69) | 0.41 |

Note: Data are the number (percentage) of children unless otherwise indicated. Percentages may not equal 100 because of rounding and missingness.

Abbreviations: IQR, interquartile range; RSV, respiratory syncytial virus; RV, rhinovirus.

**^*^** Tested by the Fisher exact test, unless otherwise indicated.

**^†^** Tested by the Wilcoxon rank-sum test.

^‡^ Defined as the use of systemic corticosteroids before the index hospitalization.

^§^ Defined as the use of systemic corticosteroids for breathing problems that caused the index hospitalization.

^¶^ Adenovirus, bocavirus, *Bordetella pertussis*, enterovirus, human coronavirus NL63, OC43, 229E, or HKU1, human metapneumovirus, influenza A or B virus, *Mycoplasma pneumoniae*, and parainfluenza virus 1–3.

**^**^** Defined as the use of invasive and/or non-invasive mechanical ventilation (e.g., continuous positive airway pressure ventilation) during the index hospitalization.

**^††^** Defined as the use of positive pressure ventilation and/or admission to intensive care unit.

# Table S3. Comparisons between male and female sexes

| **Variables** | **Overall**  **(n = 398)** | **Male sex**  **(n = 233; 59%)** | **Female sex**  **(n = 165; 41%)** | **p value^*^** |
| --- | --- | --- | --- | --- |
| Demographics |  |  |  |  |
| Age, month |  |  |  | 0.32 |
| <2 | 120 (30) | 64 (27) | 56 (34) |  |
| 2–5 | 139 (35) | 87 (37) | 52 (32) |  |
| 5–11.9 | 139 (35) | 82 (35) | 57 (35) |  |
| Race/ethnicity |  |  |  | 0.58 |
| Non-Hispanic white | 161 (40) | 98 (42) | 63 (38) |  |
| Non-Hispanic black | 91 (23) | 49 (21) | 42 (25) |  |
| Hispanic | 129 (32) | 79 (34) | 50 (30) |  |
| Other | 17 (4) | 7 (3) | 10 (6) |  |
| C-section delivery | 138 (35) | 89 (38) | 49 (30) | 0.086 |
| Prematurity (32–36.9 weeks) | 72 (18) | 46 (20) | 26 (16) | 0.36 |
| History of eczema | 61 (15) | 34 (15) | 27 (16) | 0.67 |
| Previous breathing problems (count) |  |  |  | 0.47 |
| 0 | 314 (79) | 178 (76) | 136 (82) |  |
| 1 | 62 (16) | 41 (18) | 21 (13) |  |
| ≥2 | 22 (6) | 14 (6) | 8 (5) |  |
| Ever attended daycare | 95 (24) | 55 (24) | 40 (24) | 0.91 |
| Cigarette smoke exposure at home | 60 (15) | 33 (14) | 27 (16) | 0.57 |
| Mostly breastfed during 0-2.9 months | 166 (42) | 109 (47) | 57 (35) | 0.0068 |
| Lifetime antibiotics use | 126 (32) | 78 (33) | 48 (29) | 0.38 |
| Lifetime corticosteroid use^‡^ | 62 (16) | 40 (17) | 22 (13) | 0.33 |
| Recent corticosteroid use^§^ | 42 (11) | 27 (12) | 15 (9) | 0.51 |
| Maternal smoking during pregnancy | 62 (16) | 34 (15) | 28 (17) | 0.58 |
| Clinical presentation at index hospitalization |  |  |  |  |
| Weight (kg), median (IQR) | 6.2 (4.7–8.0) | 6.5 (5.1–8.2) | 5.6 (4.5–7.7) | 0.0027**^†^** |
| Respiratory rate (per minute), median (IQR) | 49 (40–60) | 50 (40–60) | 48 (40–60) | 0.50**^†^** |
| Oxygen saturation |  |  |  | 0.48 |
| <90% | 37 (10) | 20 (8) | 17 (10) |  |
| 90–93.9% | 56 (14) | 30 (13) | 26 (16) |  |
| ≥94% | 294 (74) | 177 (76) | 117 (71) |  |
| Viral testing |  |  |  |  |
| RSV | 317 (80) | 178 (76) | 139 (84) | 0.059 |
| Solo-RSV | 225 (57) | 125 (54) | 100 (61) | 0.18 |
| RSV cycle threshold values, median (IQR) | 22 (20–25) | 21 (19–25) | 22 (20–25) | 0.83**^†^** |
| RV | 86 (22) | 55 (24) | 31 (19) | 0.27 |
| Non-RSV and non-RV | 42 (11) | 27 (12) | 15 (9) | 0.51 |
| Other pathogens^¶^ | 99 (25) | 60 (26) | 39 (24) | 0.64 |
| Clinical course |  |  |  |  |
| Received antibiotics during pre-hospitalization visit | 68 (17) | 38 (16) | 30 (18) | 0.69 |
| Received corticosteroids during pre-hospitalization visit | 37 (9) | 23 (10) | 14 (8) | 0.73 |
| Positive pressure ventilation use**^**^** | 25 (6) | 10 (4) | 15 (9) | 0.060 |
| Intensive treatment use**^††^** | 71 (18) | 35 (15) | 36 (22) | 0.086 |
| Hospital length of stay (days), median (IQR) | 2 (1–3) | 2 (1–3) | 2 (1–3) | 0.64**^†^** |
| Length of hospital stay ≥2 days | 265 (67) | 163 (70) | 102 (62) | 0.11 |

Note: Data are the number (percentage) of children unless otherwise indicated. Percentages may not equal 100 because of rounding and missingness.

Abbreviations: IQR, interquartile range; RSV, respiratory syncytial virus; RV, rhinovirus.

**^*^** Tested by the Fisher exact test, unless otherwise indicated.

**^†^** Tested by the Wilcoxon rank-sum test.

^‡^ Defined as the use of systemic corticosteroids before the index hospitalization.

^§^ Defined as the use of systemic corticosteroids for breathing problems that caused the index hospitalization.

^¶^ Adenovirus, bocavirus, *Bordetella pertussis*, enterovirus, human coronavirus NL63, OC43, 229E, or HKU1, human metapneumovirus, influenza A or B virus, *Mycoplasma pneumoniae*, and parainfluenza virus 1–3.

**^**^** Defined as the use of invasive and/or non-invasive mechanical ventilation (e.g., continuous positive airway pressure ventilation) during the index hospitalization.

**^††^** Defined as the use of positive pressure ventilation and/or admission to intensive care unit.

# Table S4. Relative abundance and carriage of the 10 most abundant nasopharyngeal fungal species among infants hospitalized for bronchiolitis

| **Species** | **Phylum** | **Relative abundance*,**  **median (IQR)** | **Carriage,**  **number (%)** |
| --- | --- | --- | --- |
| *Malassezia restricta* | *Basidiomycota* | 100 (12–100) | 355 (89) |
| *Malassezia globosa* | *Basidiomycota* | 0 (0–0) | 87 (22) |
| *Cyberlindnera americana* | *Ascomycota* | 0 (0–0) | 16 (4) |
| *Saccharomyces cerevisiae* | *Ascomycota* | 0 (0–0) | 16 (4) |
| *Penicillium chrysogenum* | *Ascomycota* | 0 (0–0) | 15 (4) |
| *Malassezia sympodialis* | *Basidiomycota* | 0 (0–0) | 12 (3) |
| *Pneumocystis jirovecii* | *Ascomycota* | 0 (0–0) | 6 (2) |
| *Cladosporium sphaerospermum* | *Ascomycota* | 0 (0–0) | 5 (1) |
| *Mucor racemosus* | *Mucoromycota* | 0 (0–0) | 2 (1) |
| *Rhizopus delemar* | *Mucoromycota* | 0 (0–0) | 2 (1) |

Abbreviation: IQR, interquartile range.

* Defined by the EukFrac, %.

# Table S5. Baseline patient characteristics and clinical course of infants hospitalized for bronchiolitis using five mycotypes in the sensitivity analysis

| **Variables** | **Mycotype 1**  **(Corresponding to mycotype A)**  **(n = 186; 47%)** | **Mycotype 2**  **(Corresponding**  **to mycotype A, C, and D)**  **(n = 43; 11%)** | **Mycotype 3**  **(Corresponding**  **to mycotype B)**  **(n = 64; 16%)** | **Mycotype 4**  **(Corresponding**  **to mycotype C)**  **(n = 79; 20%)** | **Mycotype 5**  **(Corresponding**  **to mycotype D)**  **(n = 26; 7%)** | **p value^*^** |
| --- | --- | --- | --- | --- | --- | --- |
| Demographics |  |  |  |  |  |  |
| Age, month |  |  |  |  |  | 0.45 |
| <2 | 60 (32) | 9 (21) | 19 (30) | 23 (29) | 9 (35) |  |
| 2–5 | 69 (37) | 11 (26) | 22 (34) | 29 (37) | 8 (31) |  |
| 5–11.9 | 57 (31) | 23 (53) | 23 (36) | 27 (34) | 9 (35) |  |
| Male sex | 110 (59) | 27 (63) | 36 (56) | 41 (52) | 19 (73) | 0.39 |
| Race/ethnicity |  |  |  |  |  | 0.78 |
| Non-Hispanic white | 161 (40) | 73 (39) | 12 (28) | 25 (39) | 39 (49) |  |
| Non-Hispanic black | 91 (23) | 45 (24) | 14 (33) | 13 (20) | 14 (18) |  |
| Hispanic | 129 (32) | 59 (32) | 15 (35) | 23 (36) | 24 (30) |  |
| Other | 17 (4) | 9 (5) | 2 (5) | 3 (5) | 2 (3) |  |
| C-section delivery | 62 (33) | 14 (33) | 25 (39) | 30 (38) | 7 (27) | 0.80 |
| Prematurity (32–36.9 weeks) | 38 (20) | 5 (12) | 12 (19) | 14 (18) | 3 (12) | 0.68 |
| History of eczema | 28 (15) | 11 (26) | 8 (12) | 10 (13) | 4 (15) | 0.42 |
| Previous breathing problems (count) |  |  |  |  |  | 0.035 |
| 0 | 153 (82) | 24 (56) | 51 (80) | 64 (81) | 22 (85) |  |
| 1 | 25 (13) | 13 (30) | 10 (16) | 12 (15) | 2 (8) |  |
| ≥2 | 8 (4) | 6 (14) | 3 (5) | 3 (4) | 2 (8) |  |
| Ever attended daycare | 48 (26) | 10 (23) | 15 (23) | 16 (20) | 6 (23) | 0.93 |
| Cigarette smoke exposure at home | 22 (12) | 8 (19) | 14 (22) | 12 (15) | 4 (15) | 0.33 |
| Mostly breastfed during 0­-2.9 months | 74 (40) | 14 (33) | 29 (45) | 38 (48) | 11 (42) | 0.87 |
| Lifetime antibiotics use | 56 (30) | 17 (40) | 18 (28) | 29 (37) | 6 (28) | 0.48 |
| Lifetime corticosteroid use^‡^ | 22 (12) | 8 (19) | 12 (19) | 18 (23) | 2 (8) | 0.14 |
| Recent corticosteroid use^§^ | 16 (9) | 6 (14) | 7 (11) | 13 (16) | 0 (0) | 0.10 |
| Maternal smoking during pregnancy | 29 (16) | 7 (16) | 10 (16) | 15 (19) | 1 (4) | 0.50 |
| Clinical presentation at index hospitalization |  |  |  |  |  |  |
| Weight (kg), median (IQR) | 5.8 (4.7–7.7) | 7.0 (5.7–8.9) | 6.2 (4.9–8.1) | 6.3 (4.7–7.9) | 5.7 (4.4–8.3) | 0.15**^†^** |
| Respiratory rate (per minute), median (IQR) | 48 (40–60) | 52 (40–65) | 48 (40–60) | 52 (40–60) | 54 (46–60) | 0.87**^†^** |
| Oxygen saturation |  |  |  |  |  | 0.76 |
| <90% | 15 (8) | 6 (14) | 6 (9) | 9 (11) | 1 (4) |  |
| 90–93.9% | 137 (74) | 32 (74) | 47 (73) | 60 (76) | 18 (69) |  |
| ≥94% | 27 (15) | 5 (12) | 9 (14) | 8 (10) | 7 (27) |  |
| Fungus clusters |  |  |  |  |  | 0.00050 |
| Cluster 1 (*M. restricta*-dominant) | 186 (100) | 26 (60) | 61 (95) | 0 (0) | 0 (0) |  |
| Cluster 2 (*M. globosa*-dominant) | 0 (0) | 8 (19) | 0 (0) | 79 (100) | 0 (0) |  |
| Cluster 3 (without detectable fungi) | 0 (0) | 9 (21) | 3 (5) | 0 (0) | 26 (100) |  |
| Viral testing |  |  |  |  |  |  |
| RSV | 158 (85) | 0 (0) | 64 (100) | 70 (89) | 25 (96) | <0.0001 |
| Solo-RSV | 107 (58) | 0 (0) | 51 (80) | 53 (67) | 14 (54) | 0.00050 |
| RSV cycle threshold values, median (IQR) | 21 (19–22) | Not measured | 26 (25–29) | 22 (20–24) | 21 (19–21) | <0.0001**^†^** |
| RV | 57 (31) | 1 (2) | 0 (0) | 19 (24) | 9 (35) | 0.00050 |
| Non-RSV and non-RV | 0 (0) | 42 (98) | 0 (0) | 0 (0) | 0 (0) | 0.00050 |
| Other pathogens^¶^ | 40 (22) | 33 (77) | 12 (19) | 10 (13) | 4 (15) | 0.00050 |
| Clinical course |  |  |  |  |  |  |
| Antibiotics use during pre-hospitalization visit | 31 (17) | 8 (19) | 15 (23) | 9 (11) | 5 (19) | 0.42 |
| Corticosteroids use during pre-hospitalization visit | 20 (11) | 7 (16) | 6 (9) | 4 (5) | 0 (0) | 0.12 |
| Positive pressure ventilation use**^**^** | 12 (6) | 3 (7) | 5 (8) | 1 (1) | 4 (15) | 0.081 |
| Intensive treatment use**^††^** | 25 (13) | 10 (23) | 13 (20) | 16 (20) | 7 (27) | 0.21 |
| Hospital length of stay (days), median (IQR) | 2 (1–3) | 2 (1–3) | 2 (2–3) | 2 (1–3) | 2 (1–5) | 0.35**^†^** |
| Hospital length of stay ≥2 days | 118 (63) | 31 (72) | 50 (78) | 47 (59) | 19 (73) | 0.094 |

Note: Data are the number (percentage) of children unless otherwise indicated. Percentages may not equal 100 because of rounding and missingness.

Abbreviations: IQR, interquartile range; RSV, respiratory syncytial virus; RV, rhinovirus.

**^*^** Tested by the Fisher exact test, unless otherwise indicated.

**^†^** Tested by the Kruskal-Wallis test.

^‡^ Defined as the use of systemic corticosteroids before the index hospitalization.

^§^ Defined as the use of systemic corticosteroids for breathing problems that caused the index hospitalization.

^¶^ Adenovirus, bocavirus, *Bordetella pertussis*, enterovirus, human coronavirus NL63, OC43, 229E, or HKU1, human metapneumovirus, influenza A or B virus, *Mycoplasma pneumoniae*, and parainfluenza virus 1–3.

**^**^** Defined as the use of invasive and/or non-invasive mechanical ventilation (e.g., continuous positive airway pressure ventilation) during the index hospitalization.

**^††^** Defined as the use of positive pressure ventilation and/or admission to intensive care unit.

# Figure S1. Path plot to identify an optimal number of the fungus clusters


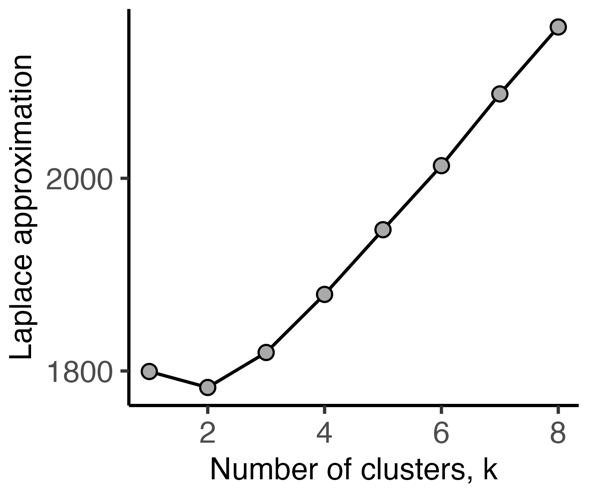


The path plot visualizes model fit across the different numbers of clusters (k of 1–8) by Dirichlet multinomial mixtures model using nasopharyngeal fungus data where at least one fungal species was detected (n=360). The model fit was evaluated by a Laplace approximation to negative log model evidence. The minimum Laplace approximation for all clusters suggests optimal fit, indicating optimal fit with k = 2.

# Figure S2. Consensus matrices, cumulative distribution function plot, and cluster consensus plots to identify an optimal number of the virus clusters

­­
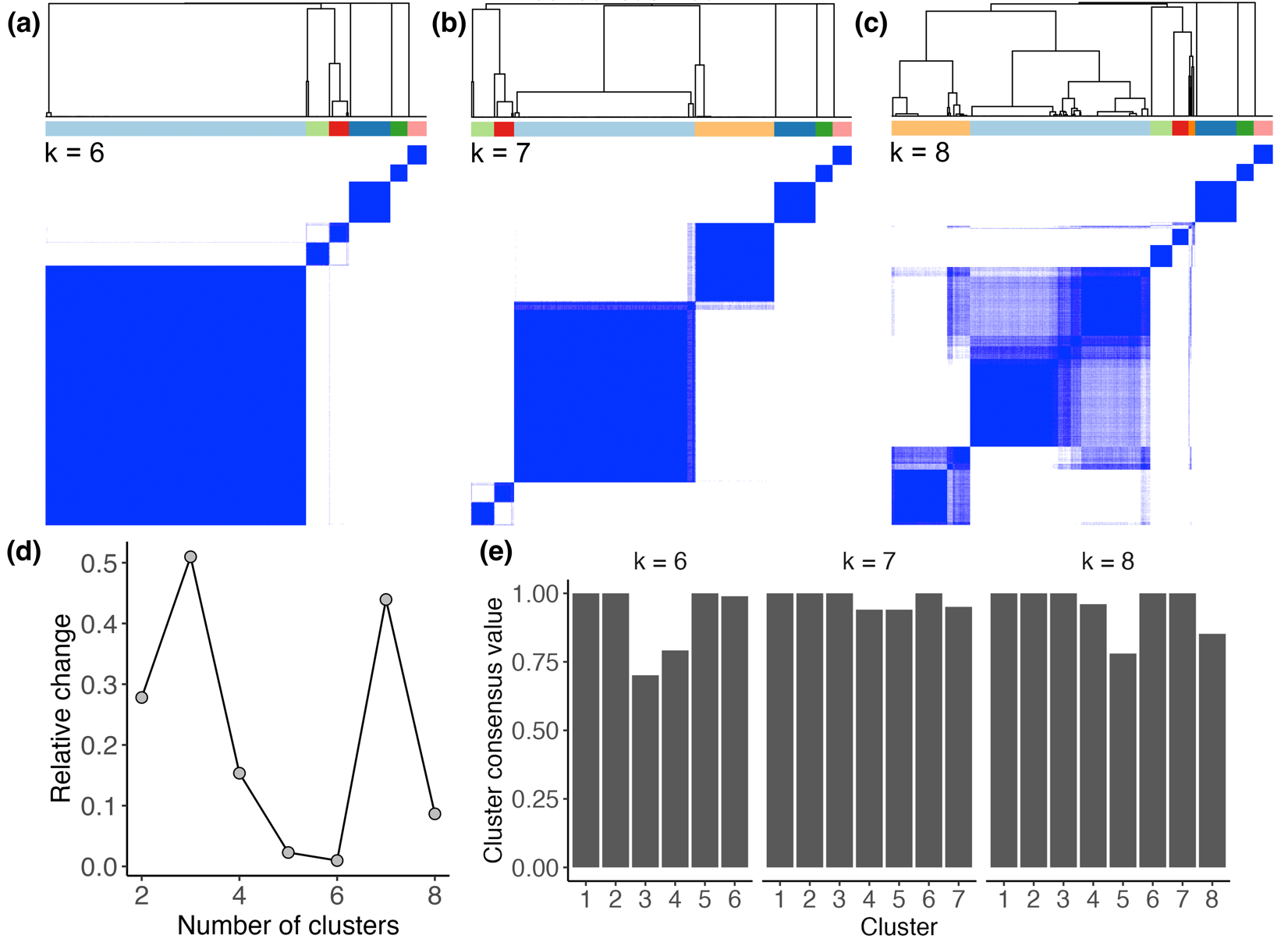


**(a-c)** The heatmaps visualize the consensus matrices for k = 6–8. The consensus matrix is obtained by taking the average over the connectivity matrices of every perturbed dataset. Consensus values range from 0 (never clustered together) to 1 (always clustered together) are marked by white to dark blue. It shows optimal partitioning in the consensus matrix with k = 7.

**(d)** The path plot visualizes the relative change in area under the cumulative distribution function (CDF) curve. Across the different numbers of clusters (k of 2–8), the relative change in the area under the CDF curve had little changes beyond k = 7.

**(e)** The bar plots show the cluster consensus value of clusters for k = 6–8. High values indicate that a cluster has high stability. Greater values for all clusters suggest optimal fit, indicating optimal fit with k = 7.

# Figure S3. Consensus matrices, cumulative distribution function plot, and cluster consensus plots to identify an optimal number of the fungus-virus clusters


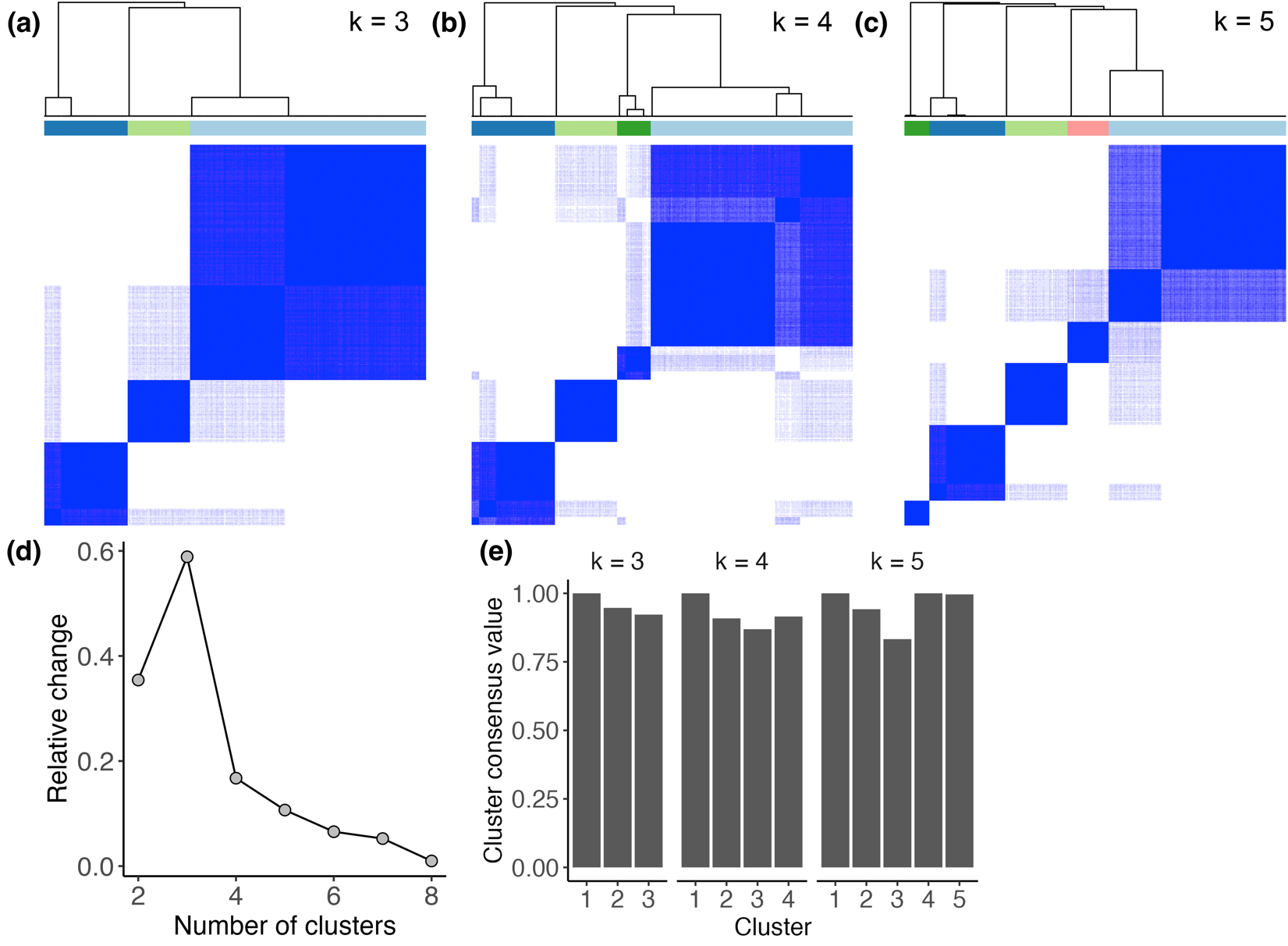


**(a-c)** The heatmaps visualize the consensus matrices for k = 3–5. The consensus matrix is obtained by taking the average over the connectivity matrices of every perturbed dataset. Consensus values range from 0 (never clustered together) to 1 (always clustered together) are marked by white to dark blue. It shows optimal partitioning in the consensus matrix with k = 4.

**(d)** The path plot visualizes the relative change in area under the cumulative distribution function (CDF) curve. Across the different numbers of clusters (k of 2–8), the relative change in the area under the CDF curve had little changes beyond k = 4.

**(e)** The bar plots show the cluster consensus value of clusters for k = 3–5. High values indicate that a cluster has high stability. Greater values for all clusters suggest optimal fit, indicating optimal fit with k = 4.

# Figure S4. Causal directed acyclic graph


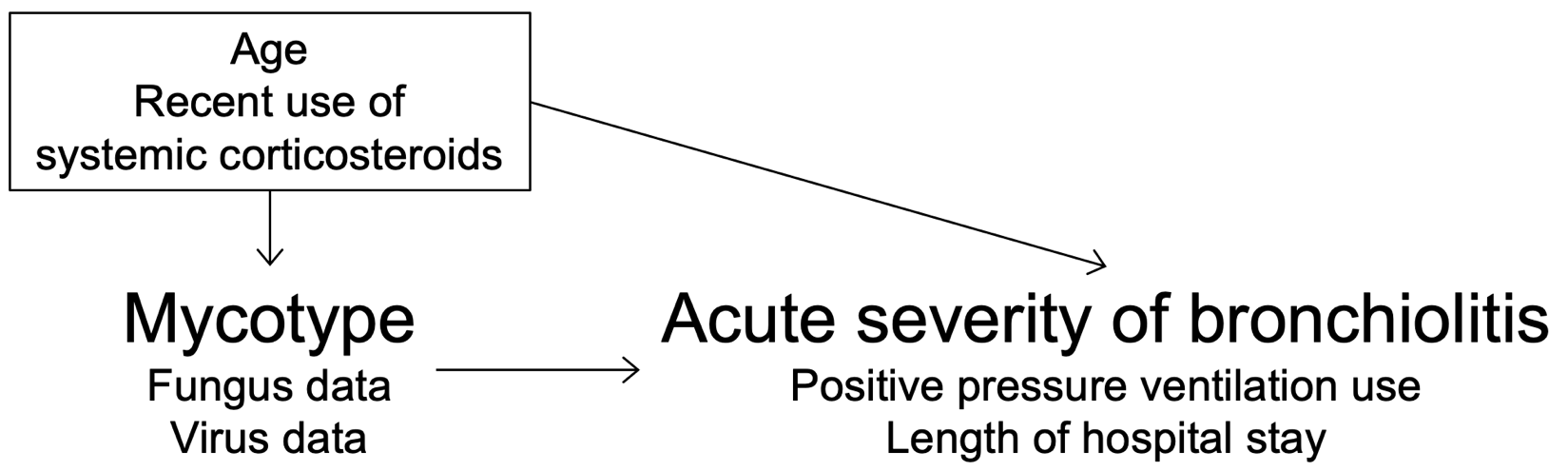


The causal directed acyclic graph was constructed to represent our proposed causal structure hypothesis linking mycotype (i.e., the exposure) and acute severity of bronchiolitis (i.e., the outcomes) with the potential confounders (in the rectangle) listed above.

# Figure S5. Association of mycotypes of infant bronchiolitis with risk of hospital length of stay of ≥2 days


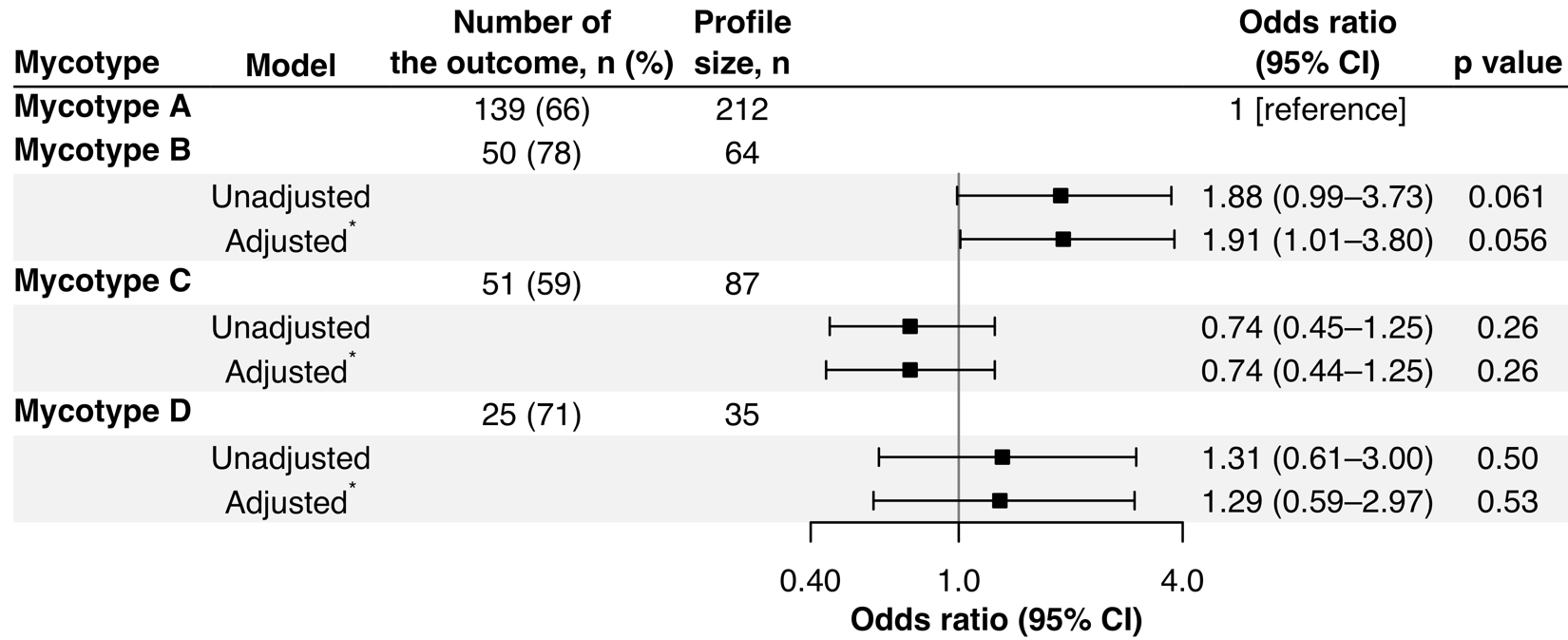


To examine the association of bronchiolitis mycotypes (mycotype A as the reference) with the risk of a hospital length of stay of ≥2 days, logistic regression models were constructed.

* Multivariable logistic regression model adjusted for potential confounders (i.e., age and use of systemic corticosteroids for breathing problems that caused the index hospitalization).

# Figure S6. Between-mycotype difference in Shannon index and relative abundance of the 25 most abundant nasopharyngeal bacterial species among infants hospitalized for bronchiolitis

**
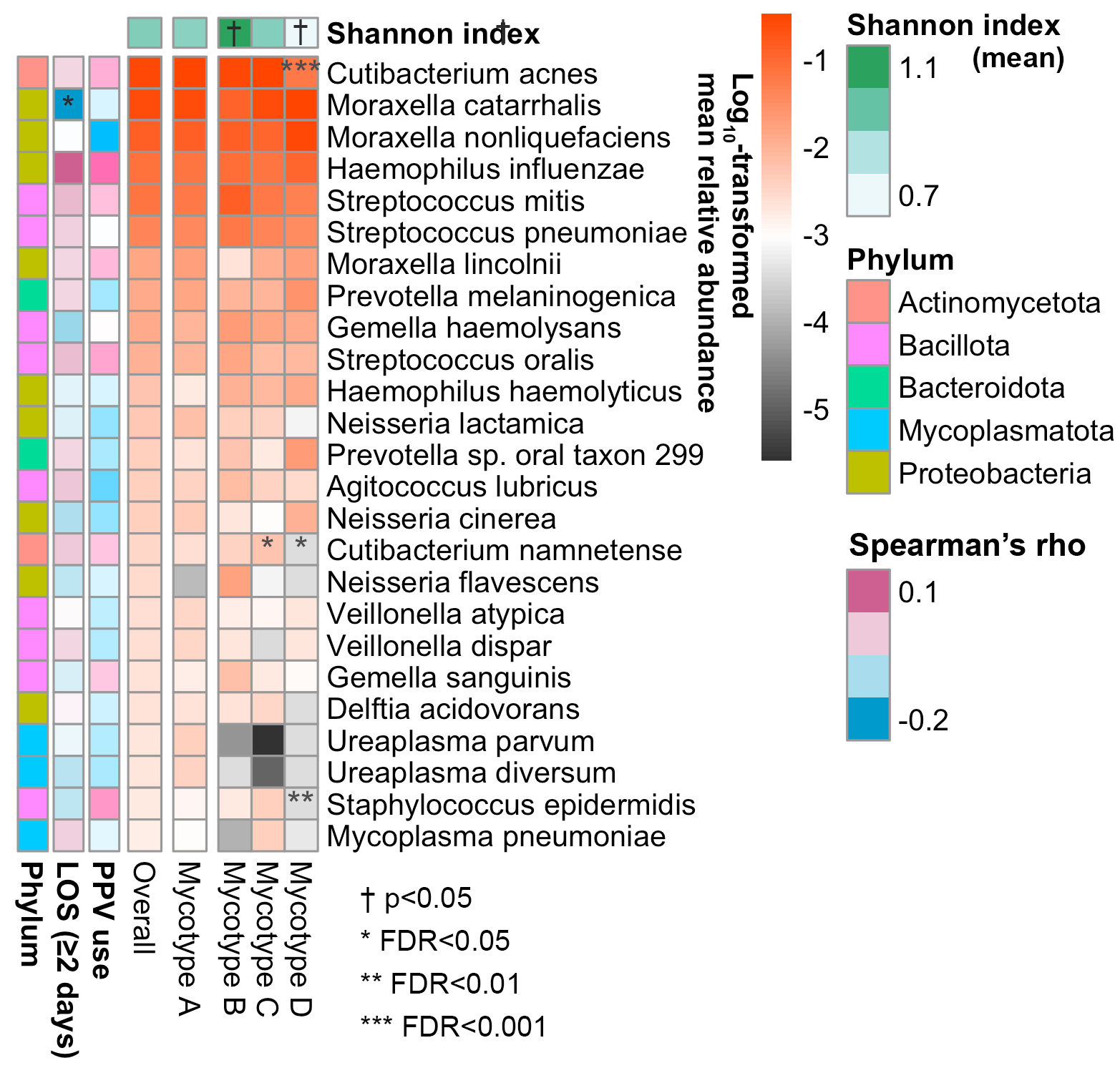
**

To examine the difference in the nasopharyngeal bacterial characteristics between mycotypes (A [the reference] vs. B, C, and D), we created the heatmap by the Shannon index and relative abundance of the 25 most abundant bacterial species using the metatranscriptome data, according to the four mycotypes. Gray cells indicate that the bacterial species were not identified among infants with the mycotype. The between-mycotype differences in the Shannon index and relative abundance compared to mycotype A were tested by the Wilcoxon rank-sum test. The relationships between the relative abundance and the binary outcome data (PPV use and hospital length of stay [LOS] of ≥2 days) were examined by using the Spearman rank correlation test.

Abbreviations: FDR, false discovery rate; PPV, positive pressure ventilation.

# Figure S7. Differential host gene expression analysis between mycotypes in infants hospitalized for bronchiolitis


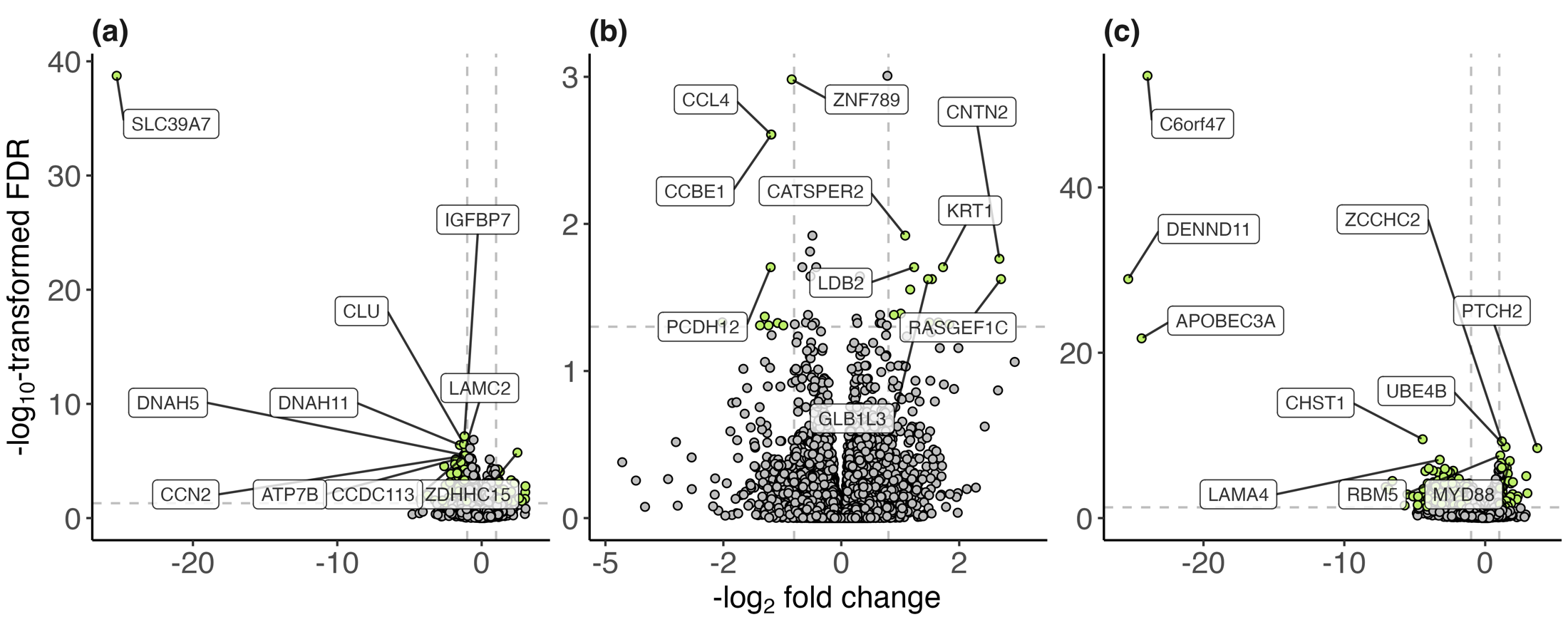


The volcano plots visualize differentially expressed host genes between mycotype **(a)** A vs. B (mycotype A was set as the reference), **(b)** A vs. C, and **(c)** A vs. D. The threshold of log_2_ fold change is |1.0| shown by the vertical dashed line, and that of false discovery rate (FDR) is < 0.05 shown by the horizontal dashed line. Genes that satisfied these two thresholds were colored green, while others were colored gray. Of the green-colored gene, the 10 genes with the lowest FDR were labeled.

# Figure S8. Between-mycotype differences in nasopharyngeal host transcriptome pathways (Gene Ontology) among infants hospitalized for bronchiolitis


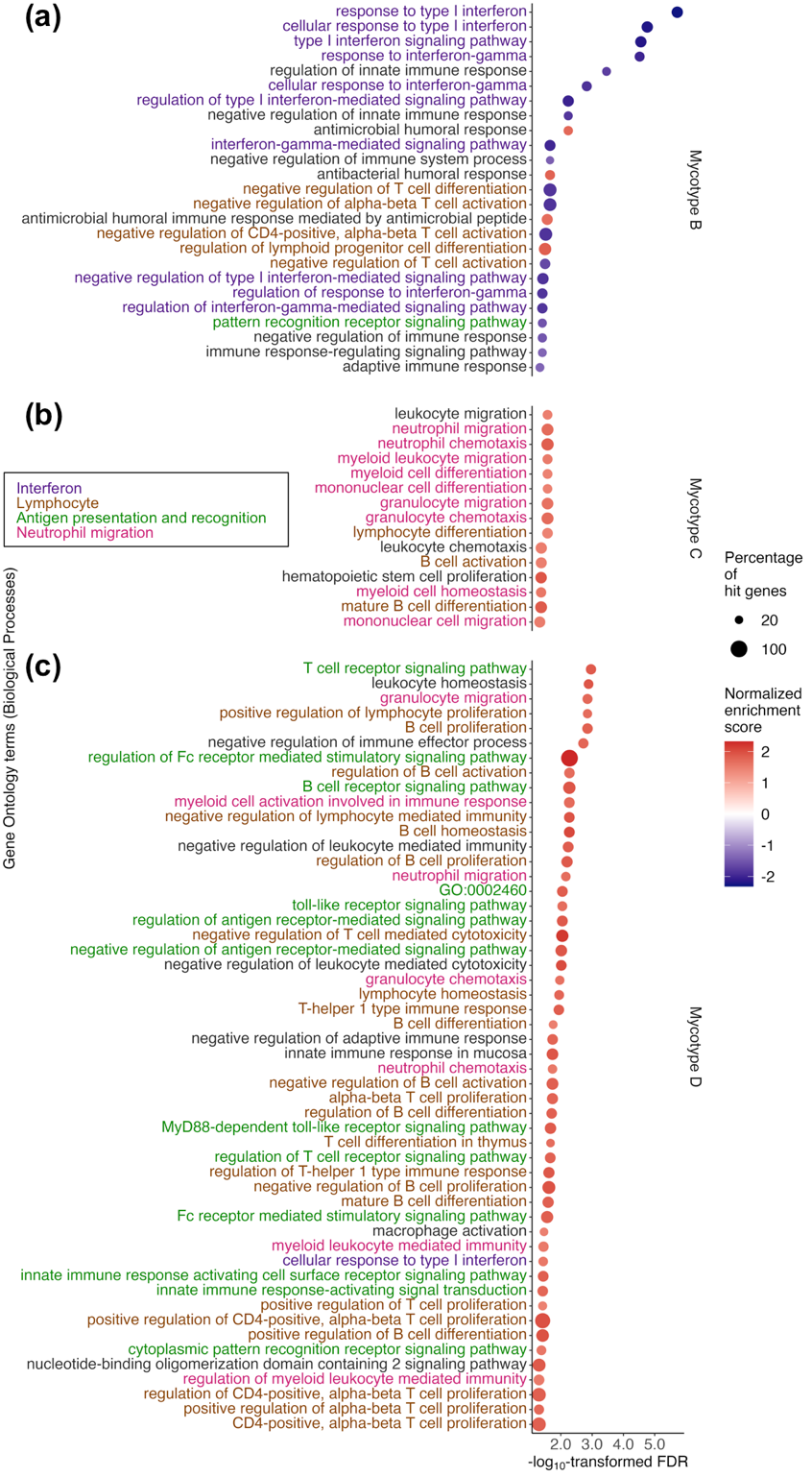


To examine the difference in the host response in the airway between mycotypes (A [the reference] vs. B, C, and D), the gene set enrichment analysis based on Biological Processes in Gene Ontology is applied to the nasopharyngeal host transcriptome data. Of the descendants of GO:0002376 immune system process, pathways with false discovery rate (FDR) <0·05 were selected for **(a)** mycotype A vs. B, **(b)** A vs. C, and **(c)** A vs. D.

Abbreviations: CD4, cluster of differentiation 4; GO:0002460, adaptive immune response based on somatic recombination of immune receptors built from immunoglobulin superfamily domains.

Figure S9. Sensitivity analysis: Association of mycotypes of infant bronchiolitis with risk of positive pressure ventilation use by logistic regression models with log*F*-type penalized method

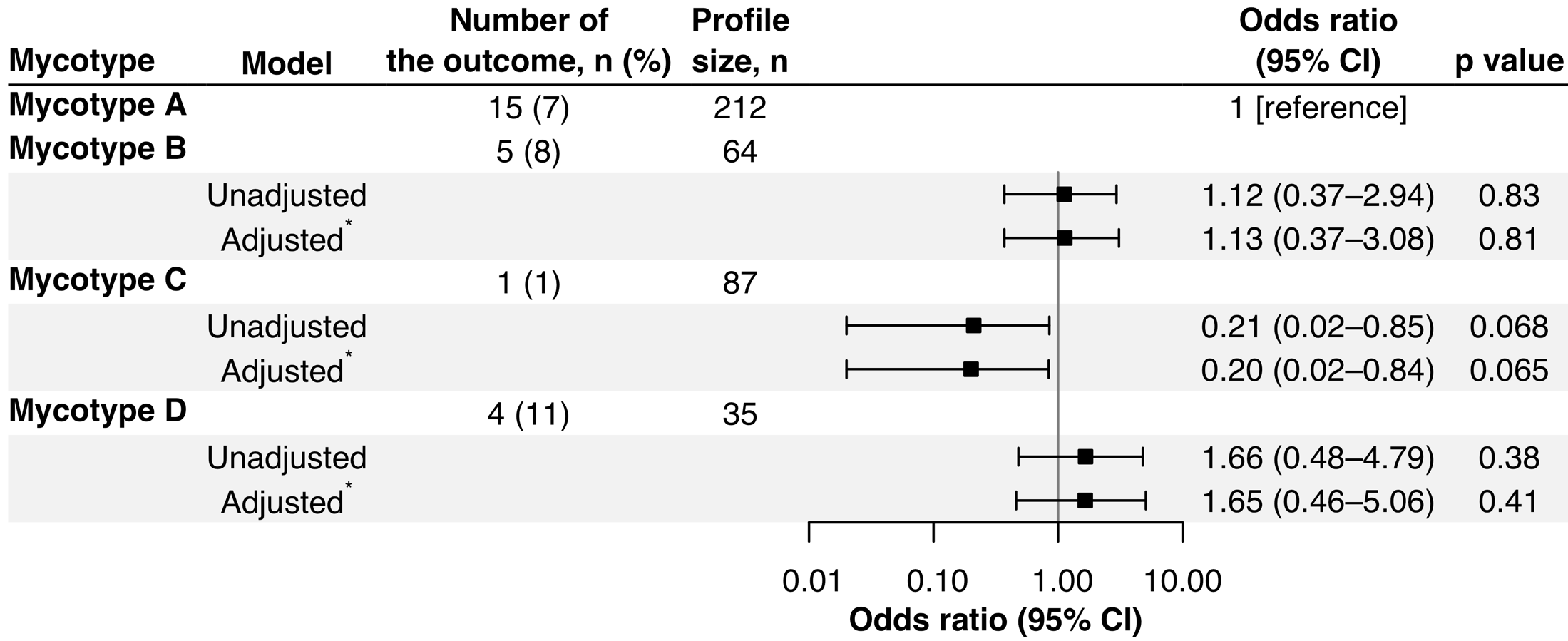


To examine the association of bronchiolitis mycotypes (mycotype A as the reference) with the risk of positive pressure ventilation use, logistic regression models were constructed. The models were corrected by log*F*-type penalized method to reduce a bias of maximum likelihood estimate for a low proportion of the outcome.

* Multivariable logistic regression model adjusted for potential confounders (i.e., age and use of systemic corticosteroids for breathing problems that caused the index hospitalization).

Figure S10. Sensitivity analysis: Association of mycotypes of infant bronchiolitis with risk of positive pressure ventilation use by modified Poisson regression models


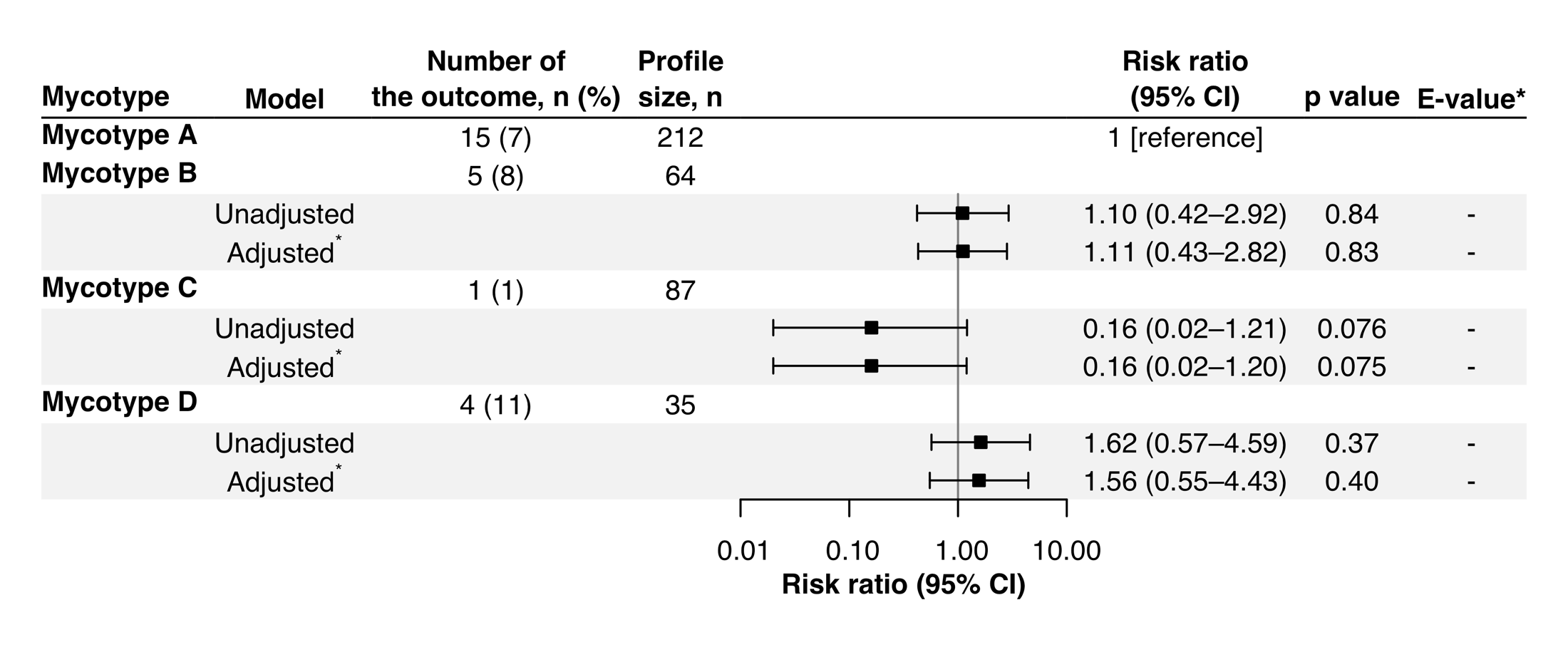


To examine the association of bronchiolitis mycotypes (mycotype A as the reference) with the risk of positive pressure ventilation use, modified Poisson regression models were constructed.

* Multivariable logistic regression model adjusted for potential confounders (i.e., age and use of systemic corticosteroids for breathing problems that caused the index hospitalization).

Figure S11. Sensitivity analysis: Association of mycotypes of infant bronchiolitis with risk of positive pressure ventilation use, limiting to infants without a previous breathing problem

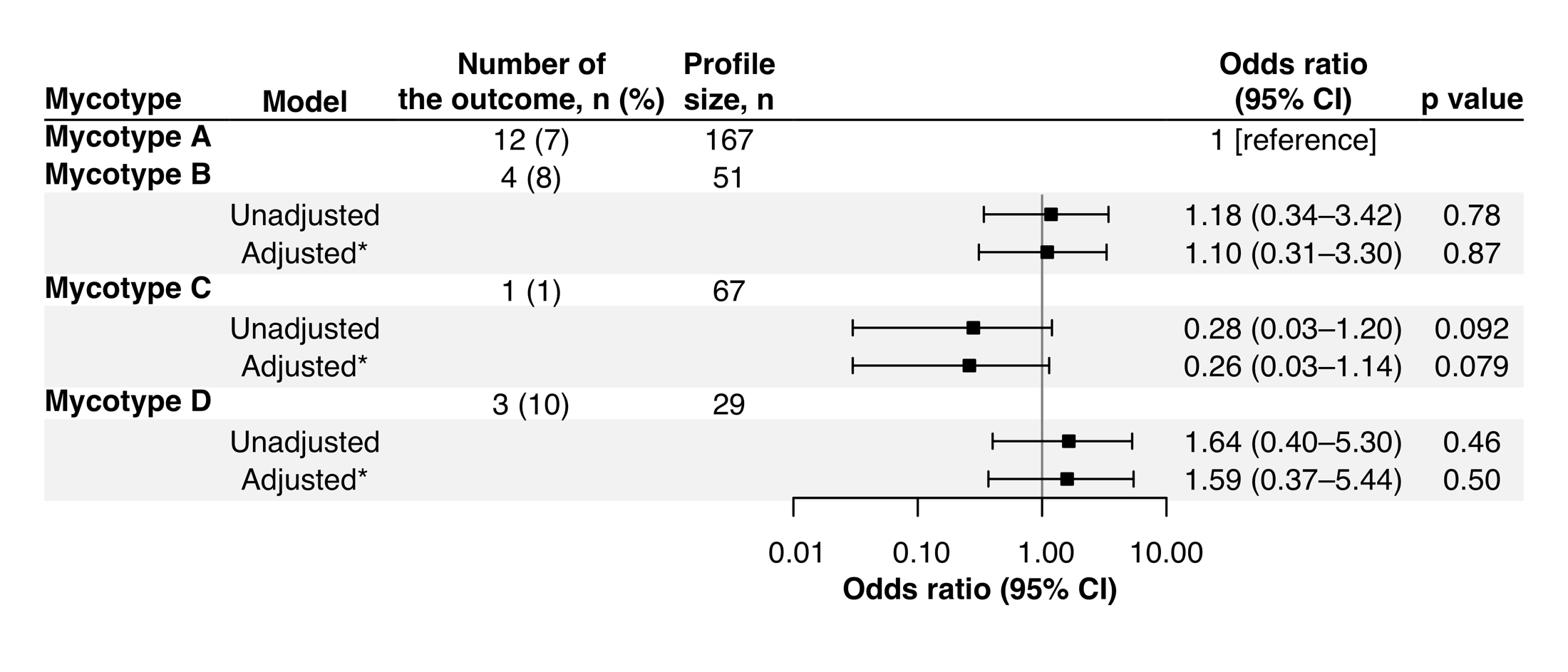


To examine the association of bronchiolitis mycotypes (mycotype A as the reference) with the risk of positive pressure ventilation use, logistic regression models were constructed. The models were corrected by Firth’s method to reduce a bias of maximum likelihood estimate for a low proportion of the outcome.

* Multivariable logistic regression model adjusted for potential confounders (i.e., age and use of systemic corticosteroids for breathing problems that caused the index hospitalization).

# Figure S12. Alluvial plot to examine consistencies across different numbers of mycotypes


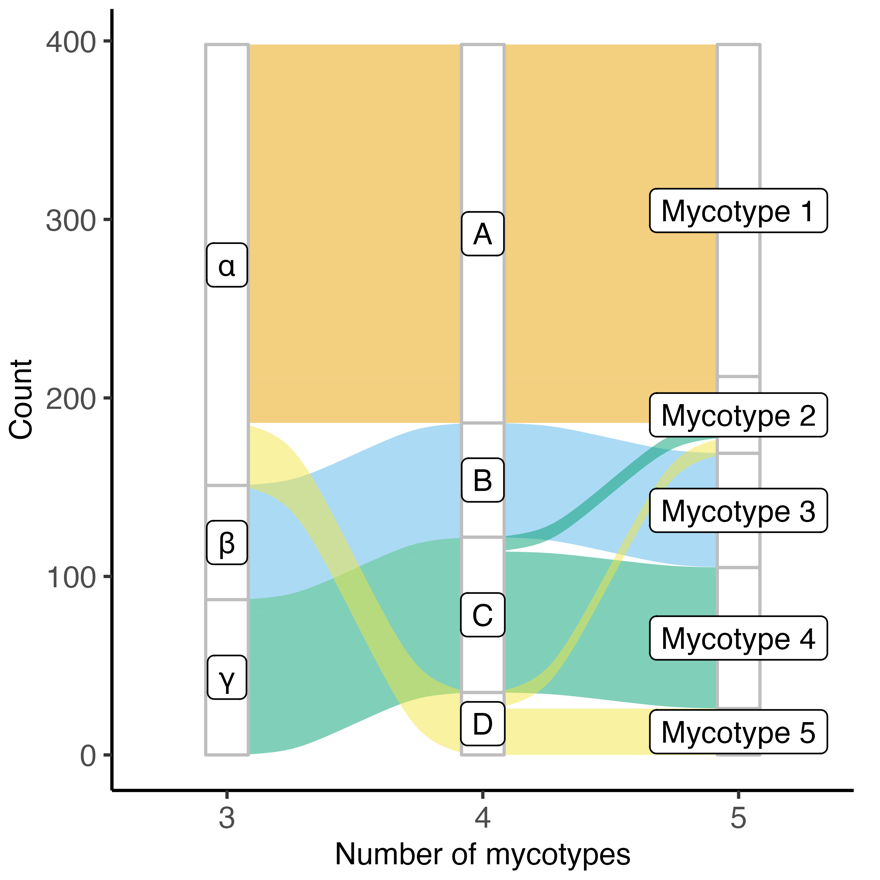


The *X* axis indicates the number of mycotypes (range of 3–5); the *Y* axis indicates the count of infants. Each color band represents a group of infants with an original mycotype (A–D). Consistencies were confirmed between the four original mycotypes (A–D) and three and five mycotypes.

# Figure S13. Sensitivity analysis: Clinical, virus, and fungus characteristics of infants hospitalized for bronchiolitis, according to mycotypes, by using five mycotypes


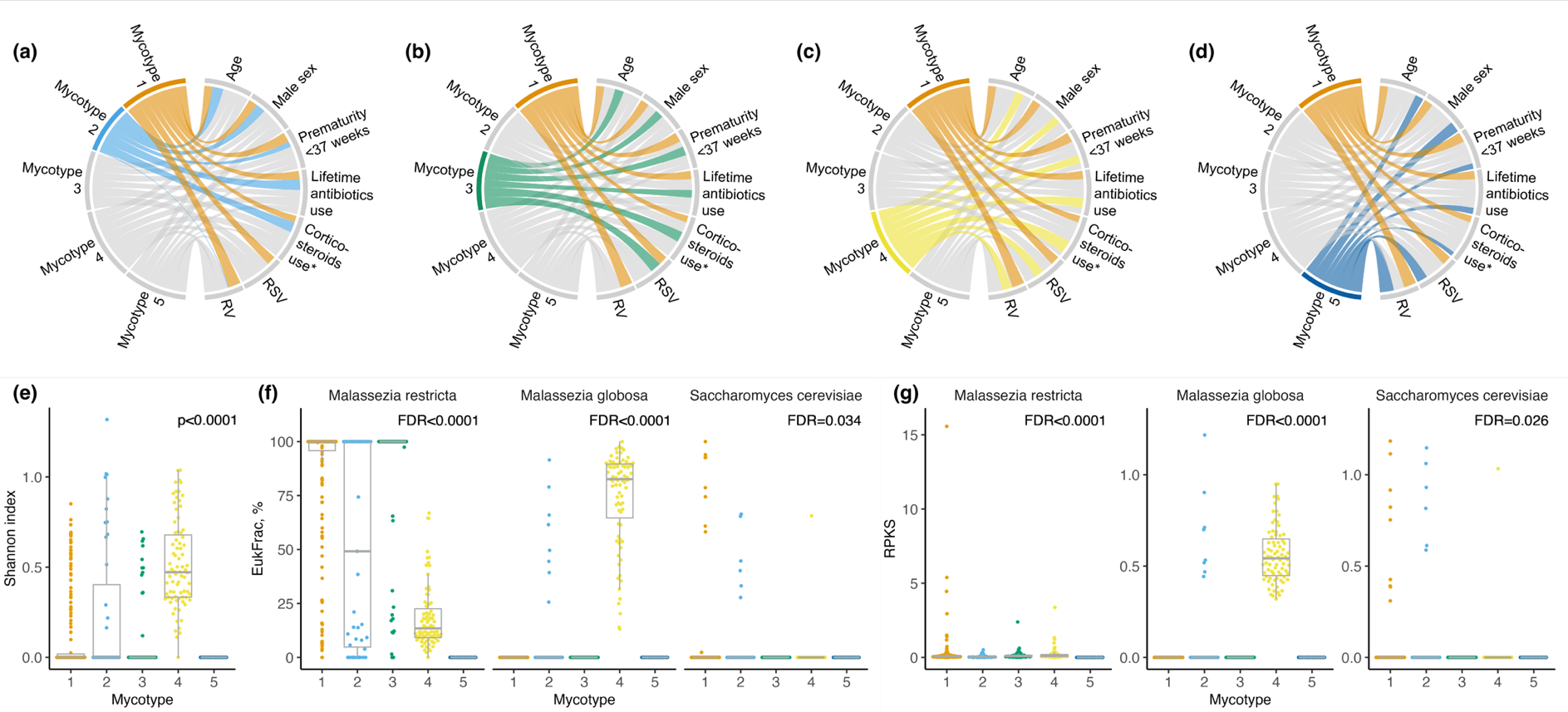


To interpret clinical and virus characteristics of the five mycotypes, we constructed chord diagrams that represent the comparison between mycotypes **(a)** 1 and 2, **(b)** 1 and 3, **(c)** 1 and 4, and **(d)** 1 and 5. Ribbons connect each of the mycotypes (mycotypes 1–5) with major clinical and virus characteristics. The width of the ribbon represents the proportion of infants within the mycotypes who have the corresponding clinical or virus characteristic, which was scaled to a total of 100%. Additionally, to interpret fungus characteristics, we constructed bee swarm and box-and-whisker plots to show the distribution of **(e)** the Shannon index as well as **(f)** EukFrac (i.e., relative abundance) and **(g)** reads per kilobase of sequence (RPKS; i.e., absolute abundance) of the three most abundant nasopharyngeal fungal species, according to the five mycotypes. In the box-and-whisker plots, boxes and whiskers show the median and interquartile range and 1.5 times the IQR, respectively. In the overlying bee swarm plots, each point denotes each infant, and the width represents the probability that infants in a mycotype take on a specific value. The between-mycotype differences in the Shannon index, EukFrac, and RPKS were tested by the Kruskal-Wallis test.

Abbreviations: RSV, respiratory syncytial virus; RV, rhinovirus.

* Defined as the use of systemic corticosteroids for breathing problems that caused the index hospitalization.

# Figure S14. Sensitivity analysis: Association of mycotypes of infant bronchiolitis with risk of positive pressure ventilation use, using five mycotypes


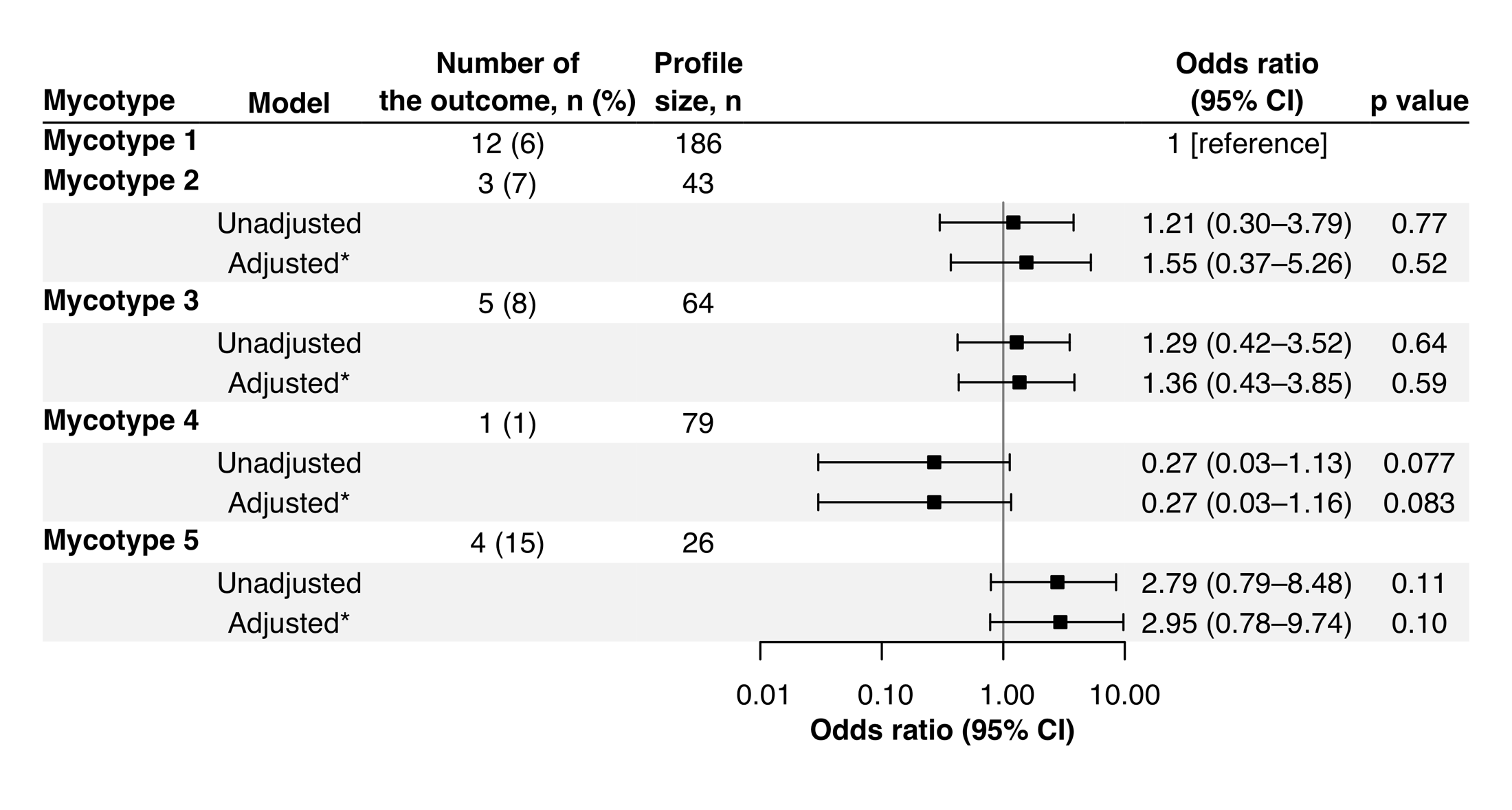


To examine the association of bronchiolitis mycotypes (mycotype 1 as the reference) with the risk of positive pressure ventilation use, logistic regression models were constructed. The models were corrected by Firth’s method to reduce a bias of maximum likelihood estimate for a low proportion of the outcome.

* Multivariable logistic regression model adjusted for potential confounders (i.e., age and use of systemic corticosteroids for breathing problems that caused the index hospitalization).
